# Clinical and Genetic Predictive Models for the Prediction of Pathological Complete Response to Optimize the Effectiveness for Trastuzumab Based Chemotherapy

**DOI:** 10.3389/fonc.2021.592393

**Published:** 2021-07-15

**Authors:** Lun Li, Min Chen, Shuyue Zheng, Hanlu Li, Weiru Chi, Bingqiu Xiu, Qi Zhang, Jianjing Hou, Jia Wang, Jiong Wu

**Affiliations:** ^1^ Department of Breast Surgery, Shanghai Cancer Center, Fudan University, Shanghai, China and Department of Oncology, Shanghai Medical College, Fudan University, Shanghai, China; ^2^ Collaborative Innovation Center for Cancer Medicine, Shanghai, China

**Keywords:** breast cancer, HER2, neoadjuvant chemotherapy, predictive model, immune signature, trastuzumab

## Abstract

**Background:**

Trastuzumab shows excellent benefits for HER2+ breast cancer patients, although 20% treated remain unresponsive. We conducted a retrospective cohort study to optimize neoadjuvant chemotherapy and trastuzumab treatment in HER2+ breast cancer patients.

**Methods:**

Six hundred patients were analyzed to identify clinical characteristics of those not achieving a pathological complete response (pCR) to develop a clinical predictive model. Available RNA sequence data was also reviewed to develop a genetic model for pCR.

**Results:**

The pCR rate was 39.8% and pCR was associated with superior disease free survival and overall survival. ER negativity and PR negativity, higher HER2 IHC scores, higher Ki-67, and trastuzumab use were associated with improved pCR. Weekly paclitaxel and carboplatin had the highest pCR rate (46.70%) and the anthracycline+taxanes regimen had the lowest rate (11.11%). Four published GEO datasets were analyzed and a 10-gene model and immune signature for pCR were developed. Non-pCR patients were ER^+^PR^+^ and had a lower immune signature and gene model score. Hormone receptor status and immune signatures were independent predictive factors of pCR.

**Conclusion:**

Hormone receptor status and a 10-gene model could predict pCR independently and may be applied for patient selection and drug effectiveness optimization.

## Introduction

Breast cancer is one of the most common cancers and the leading cause of cancer death among females all over the world (1). HER2 gene amplification or protein overexpression accounts for approximately 20% of invasive breast cancer cases (2). In the absence of HER2-targeted therapies, HER2 positivity is associated with a worse prognosis (3). Although targeted therapy is currently readily accessible, patients with large tumor size and clinically positive lymph nodes still exhibit the worst prognosis. Neoadjuvant chemotherapy and trastuzumab (NACT), an approach whereby patients receive preoperative treatments, has been a standard treatment strategy for locally advanced breast cancer. NACT could allow for treatment response monitoring by measuring changes in tumor size (4). Administering NACT to patients may increase the possibility of surgery with negative margins by downsizing the tumor stage and may further increase the probability of achieving a pathological complete response (pCR), which could be translated into better long-term survival (5). Several clinical trials have confirmed improved survival in HER2+ breast cancer patients who achieved pCR (4, 6–8). However, there was a high proportion of HER2+ breast cancer patients who failed to respond. Thus, better characterization of these patients might be helpful for improving their prognosis. To select patients most likely not to respond to NACT and to choose optimal treatment regimens, the characteristics of those who did not achieve pCR should be well described and models for the prediction of response to NACT are required.

Thus, in the present study, we aimed to summarize the clinical and genetic characteristics of patients who did not achieve pCR among HER2+ breast cancer patients in the neoadjuvant setting. In addition, we aimed to develop a predictive model for pCR in order to optimize the effectiveness for trastuzumab based chemotherapy.

## Methods

This study was approved by Ethics Committee in Fudan University Shanghai Cancer Center (FUSCC). Written consent for study participation was waived due to the retrospective nature of the study.

### Inclusion Criteria for the Real-World Cohort

Only those patients who were HER2-positive (HER2 3+ by immunohistochemistry (IHC), or fluorescence *in situ* hybridization (FISH) positive) breast cancer who received neoadjuvant chemotherapy with or without trastuzumab were included. Neoadjuvant chemotherapy was defined as chemotherapy before surgery (breast and axillary surgery). We excluded those patients in which all breast cancer tissue was resected before chemotherapy as for these patients, judging postoperative chemotherapy response is impossible, and those with distant metastasis (M1) confirmed by biology.

### Clinical Variables and Cohort

Patients were identified from the breast cancer database constructed by FUSCC. All information was retrieved from medical records, and double-checked by three authors (LL, MC, SY-Z). Prognostic information was retrieved from the breast cancer database. Our hospital has its own department to follow up the patients, and we also checked their recent visits.

The variables analyzed included the patent’s clinical stage (tumor size, nodal status), preoperative receptor (ER/PR) status, HER2 status, Ki-67 expression, neoadjuvant chemotherapy regimens, use of trastuzumab, surgery, postoperative response, pathological stage (tumor size, nodal status), and adjuvant treatments (chemotherapy, radiotherapy, and endocrine therapy). Clinical and pathological stages were classified according to the AJCC version 8 (9). Clinical stage before biology was assessed based on the data from Magnetic Resonance Imaging, ultrasonography, and physical examination. In most cases, these were consistent with each other. For those patients with inconsistent data, averaged values were used. The description of the breast tumor such as redness and swelling were also used in the clinical stage. ER and PR status were defined as positive when expression was ≥1%. Ki-67 was defined positivity if it was ≥20%. The pCR was defined as no invasive tumor or axillary lymph nodes (ypT0/is ypN0) (8). The prognostic outcomes assessed were overall survival (OS) and disease-free survival (DFS).

### Genetic Variables

The RNA sequence data from GSE37946 (10), GSE50948 (11), GSE66305 (12), and GSE130788 (13) cohorts were analyzed. The characteristics of the published cohorts were presented in **Supplemental Table 1**. The expression of each gene was normalized relative to GAPDH (Δ=log2(x/g), x: the gene expression level; g: the expression level for GAPDH) and standardized from 0 to 10 [(x-min) × 10/(max-min)]. Differentially expressed genes (DEGs) were defined with a threshold of *p*-value < 0.05. The immune signatures for B cells, T cells, natural killer (NK) cells, chemokine, metabolic, and cell proliferation pathways were built based on DEGs between pCR and non-pCR patients.

### Model Development and Validation

The clinical model for pCR was developed based on the multivariate logistic regression analysis results. The training clinical cohort was based on the retrospective analysis of all HER2+ breast cancer patients that received neoadjuvant chemotherapy with or without trastuzumab from 2012 to 2016 at our center. The validation cohort was based on patient data from 2017 in our institution. In addition, published cohorts GSE22358 (14), GSE50948 (11), and GSE130788 (13) from GEO datasets were used for the validation.

For genetic models, the same DEGs across different GEO databases were used for model development. Three databases (GSE37946, GSE50948, GSE66305) were chosen as training cohorts, and GSE130788 was used to validate the model.

### Statistical Analysis

Data was analyzed using SPSS version 21.0 (SPSS Inc., Chicago, IL, USA, version 20) and R software. Categorical variables were expressed using frequency, and continuous data was expressed using mean and standard variance, as well as median and interquartile range (IQR). The chi-squared test or Fisher’s exact test, also by univariate logistic regression analysis was used to analyze the relationship between variables and pCR. The Odds ratio (OR) with its 95% CI was calculated. Those clinical variables with significant associations in univariate analyses were further analyzed by multivariate logistic regression analyses. Sensitivity analyses were conducted to check whether the results were stable across different kinds of population. The clinical model was developed based on multivariate logistic regression analyses.

The DEGs in each dataset were analyzed using R software “limma” packages. The lasso regression methods were used for the development of the genetic models. The predictive abilities for clinical and genetic models were assessed by the Area Under Curve (AUC) value based on the receiver operating characteristic curve (ROC) analysis.

Survival was estimated by the Kaplan-Meier method. Univariate and multivariate analyses were performed using Cox proportional hazards regression and the hazard ratio (HR) with its 95% confidence interval (95% CI) was calculated. All important factors that might influence DFS and OS were considered for multivariate analysis. *p* < 0.05 was considered statistically significant.

## Results

### Characteristics of Included Patients

In the training cohort, 600 patients who received neoadjuvant trastuzumab from the FUSCC were included with a median follow-up 1484 days (IQR 1176.25-1939 days). Forty-nine patients were lost to follow-up, 60 (10%) patients died, and 112 patients (18.67%) experienced an event (relapse or metastasis). Most patients were stage II (347/600, 57.83%) and stage III (244/600, 40.67%). Specifically, most patients were staged cT2 (330/600, 55%) and cT3 (144/600, 24%), cN1 (346/600, 57.67%). There were 346 ER^+^ (346/600, 57.67%) and 404 PR^+^ (404/600, 67.33%) patients. Overall, 528 patients (88%) were scored 3+ by IHC, and 545 patients (90.83%) expressed ≥20% Ki-67. Most patients used PC (paclitaxel and carboplatin, 364/600, 60.67%) as the chemotherapy regimen and 318 (53%) patients who did not complete all predefined cycles of neoadjuvant chemotherapy. 517 patients (86.17%) received trastuzumab, 525 patients (87.5%) underwent mastectomy, and the most frequent axillary treatment was axillary lymph node dissection (ALND) (479/600, 79.83%). We developed a validation cohort using data from 2017 in our hospital, and 165 HER2+ breast cancer patients who received neoadjuvant trastuzumab were included. The characteristics of the validation cohort are listed in **Supplemental Table 2**. All patients did not receive neoadjuvant pertuzumab, lapatinib.

### pCR Was Associated With Improved Survival in HER2+ Breast Cancer Patients

In the FUSCC training cohort, the 1, 3, 5, 7-year survival rates were 96.3%, 84.2%, 79.5%, and 77.4% for DFS and 99.3%, 93.8%, 87% and 85.3% for OS, respectively. Those patients with stages cT4 (DFS: HR 2.82, 95% CI 1.14-6.98, *p*=0.03; OS: HR 3.75, 95% CI 1.07-13.16, *p*=0.04) and cN3 (DFS: HR 2.25, 95% CI 1.22-4.16, *p*=0.01; OS: HR 3.98, 95% CI 1.67-9.49, *p*=0.002) had worse survival than cT1 and cN0 (**Supplemental Tables 3**, **4**). Age, BMI, menopausal status, preoperative ER, PR, HER2 status, and higher Ki-67 were not associated with better DFS or OS.

**Figure 1:**
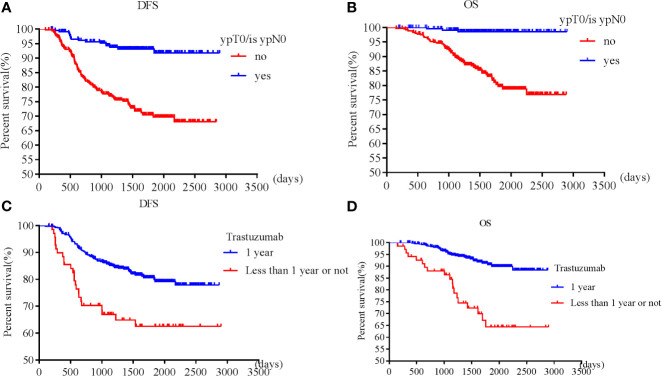
Survival according to pathological complete response [**(A)** disease free survival; **(B)** overall survival] and trastuzumab use [**(C)** disease free survival; **(D)** overall survival].

Patients with pT0 and pTis had the best and similar survival (DFS: HR 1.21, 95% CI 0.49-3.00, *p*=0.68; OS: HR 0.52, 95% CI 0.07-4.19, *p*=0.54). Postoperative pathological T and N stages were negatively associated with OS and DFS. Higher pathological T and N stages had worse OS and DFS. However, postoperative invasive ductal cancer with or without DCIS were associated with the worst survival (DFS: HR 3.39, 95% CI 2.08-5.53, *p*<0.001; HR 3.47, 95% CI 1.83-6.57, *p*<0.001; OS: HR 5.02, 95% CI 2.35-10.71, *p*<0.001; HR 5.37, 95% CI 2.12-13.63, *p*<0.001). Patients achieving pCR were associated with longer survival (DFS: HR 0.23, 95% CI 0.13-0.38, *p*<0.001; OS: HR 0.08, 95% CI 0.02-0.24, *p*<0.001) than those who did not achieve (**Figures 1A, B**).

Patients who received adequate (12 months) trastuzumab therapy experienced longer survival than those with fewer than 12 months’ treatment or those without trastuzumab treatment (DFS: HR 0.49, 95% CI 0.29-0.85, *p*=0.01; OS: HR 0.30, 95% CI 0.15-0.60, *p*=0.001) (**Figures 1C, D**). pT, pN, postoperative residual invasive tumor, pCR and trastuzumab treatment for 1 year were all independent prognostic factors for DFS and OS, while cT, cN, preoperative ER, PR, and endocrine therapy were independent prognostic factors for OS.

When analyzing those who received neoadjuvant trastuzumab, pT, pN, postoperative residual invasive tumor, and pCR were all independent prognostic factors for DFS and OS. However, only pT2 and pT3 were independently associated with shorter DFS, while pT2 was independently associated with shorter OS (**Supplemental Tables 5**, **6**). When analyzing those who received neoadjuvant wPC and trastuzumab, pN and pCR were all independent prognostic factors for DFS and OS (**Supplemental Tables 7**, **8**).

### Patient Characteristics for Non-pCR Among HER2+ Breast Cancer Patients in the Training Cohort

The pCR rate was 39.8%, and did not differ across clinical T stages (*χ* ^2^ = 3.16, *p*=0.37), N stages (*χ* ^2^ = 0.19, *p*=0.98), age (*χ* ^2^ = 0.66, *p*=0.72), BMI (*χ* ^2^ = 0.58, *p*=0.45), or menopausal status (*χ* ^2^ = 0.03, *p*=0.87) (**Table 1**). The pCR rates were lower in patients who were ER^+^ (*χ* ^2^ = 39.37, *p*<0.001) and PR^+^ (*χ* ^2^ = 32.52, *p*<0.001). IHC score of 1+ had the lowest pCR rate (*χ* ^2^ = 11.31, *p*=0.003). Lower Ki67 (<20%) was associated with lower pCR possibility (*χ* ^2^ = 3.99, *p*=0.046). Neoadjuvant trastuzumab was associated with higher pCR (*χ* ^2^ = 19.03, *p*<0.001). Those patients who completed all predefined cycles of NACT (282/600, 47%) had a higher pCR rate than those who did not (46.45% *vs*. 33.96%, *p*=0.002). Among the neoadjuvant chemotherapy regimens, weekly paclitaxel and carboplatin (wPC) had the highest pCR rate (46.70%) and the anthracycline+taxanes regimen (TAC) regimens had the lowest pCR rate (11.11%). Paclitaxel and carboplatin administrated every three weeks (3wPC) and anthracycline followed taxanes (AC-T/P) had similar pCR rates (35.53% *vs.* 33.85%). Overall, negative ER and PR status was associated with higher pCR probability.

**Table 1:** Logistic analysis for factors that affect pCR in the training cohort.

	Variables	All	Non-pCR	pCR	pCR(%)	Univariate logistic analysis		multivariate logistic analysis	
						OR(95% CI)	P-value	OR(95% CI)	P-value
cT	cT1	48	25	23	47.92%	Ref			
	cT2	330	204	126	38.18%	0.67(0.37-1.23)	0.2		
	cT3	144	82	62	43.06%	0.82(0.43-1.58)	0.56		
	cT4	67	44	23	34.33%	0.57(0.27-1.21)	0.14		
cN	cN0	150	89	61	40.67%	Ref			
	cN1	346	207	139	40.17%	0.98(0.66-1.45)	0.92		
	cN2	33	20	13	39.39%	0.95(0.44-2.05)	0.89		
	cN3	69	43	26	37.68%	0.88(0.49-1.59)	0.68		
Age	<35	58	37	21	36.21%	Ref			
	35-65	511	304	207	40.51%	1.2(0.68-2.11)	0.53		
	>65	31	20	11	35.48%	0.97(0.39-2.41)	0.95		
BMI	≥25	406	240	166	40.89%	Ref			
	<25	194	121	73	37.63%	0.87(0.61-1.24)	0.45		
Menopausal status	Yes	241	144	97	40.25%	Ref			
	No	359	217	142	39.55%	0.97(0.7-1.36)	0.86		
ER	Negative	346	171	175	50.58%	Ref			
	Positive	254	190	64	25.20%	0.33(0.23-0.47)	<0.001	0.5(0.3-0.82)	0.01
PR	Negative	404	211	193	47.77%	Ref			
	Positive	196	150	46	23.47%	0.34(0.23-0.49)	<0.001	0.53(0.31-0.92)	0.02
HER2	Her2 3+	528	305	223	42.23%	Ref			
	Her2 2+	62	47	15	24.19%	0.15(0.02-1.21)	0.08	0.32(0.04-2.7)	0.29
	Her2 1+	10	9	1	10.00%	0.44(0.24-0.8)	0.007	0.45(0.24-0.85)	0.01
Ki67	<20%	55	40	15	27.27%	Ref			
	≥20%	545	321	224	41.10%	1.86(1-3.45)	0.049	1.57(0.81-3.06)	0.18
Trastuzumab	No	83	68	15	18.07%	Ref			
	Yes	517	293	224	43.33%	3.47(1.93-6.22)	<0.001	2.44(1.25-4.79)	0.01
Enough cycle	No	318	210	108	33.96%	Ref			
	Yes	282	151	131	46.45%	1.69(1.21-2.35)	<0.001	1.5(1.02-2.21)	0.04
Chemotherapy	wPC	364	194	170	46.70%	Ref		(-)	
	3wPC/TC	76	49	27	35.53%	0.63(0.38-1.05)	0.08	0.62(0.36-1.07)	0.08
	AC-T/P	65	43	22	33.85%	0.58(0.34-1.02)	0.06	0.62(0.34-1.14)	0.12
	TAC(PAC, PEC, TEC)	18	16	2	11.11%	0.14(0.03-0.63)	0.01	0.39(0.08-2.04)	0.27
	Other	77	59	18	23.38%	0.35(0.2-0.61)	<0.001	0.42(0.22-0.78)	0.01

When analyzing those who received neoadjuvant trastuzumab, lower pCR rates were found in those who were ER^+^, PR^+^, HER2 1+, or those who did not complete all predefined cycles. Univariate and multivariate logistic analysis confirmed that ER, PR, HER2, and cycles of neoadjuvant chemotherapy were independent factors for pCR (**Supplemental Tables 9**, **10**). When analyzing those who received neoadjuvant wPC and trastuzumab, only ER and PR statuses were associated with pCR by univariate logistic analysis. But only ER status was confirmed by multivariate logistic analysis (**Supplemental Tables 11**, **12**).

**Figure 2:**
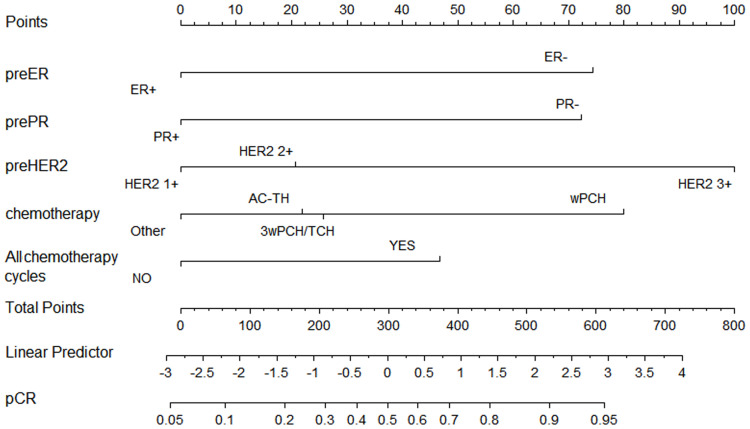
Nomogram to predict pathological complete response after preoperative chemotherapy and trastuzumab for HER2 positive breast cancer.

Based on our multivariate logistic analysis, we developed a model that consisted of preoperative ER status, PR status, and HER2 status, chemotherapy regimen types and the number of cycles for those patients who received neoadjuvant trastuzumab and chemotherapy. The formula was:

y1=-0.695×ER-0.683×PR+0.688×HER2-0.210×chemotherapy+0.388×all-cycle-1.61 (**Figure 2**, supplemental materials).

Although the AUC value was the highest for preoperative ER status (AUC = 0.63, *p <*0.001), the predictive value of this model (AUC =0.69, *p <*0.001) was higher than any of the single clinical factor (**Supplemental Figure 1**). Patients with a higher model score (>-0.5395) had a higher probability of achieving pCR in the training cohort (57.60% *vs.* 24.10%, *p <*0.001).

### Validation of Clinical Models

A validation cohort consisting of 165 patients in our institution was developed (**Supplemental Table 2**). In this validation cohort, ER negativity, PR negativity, HER2 positivity, higher Ki-67 expression, and trastuzumab use were associated with higher probability of pCR. However, in this cohort, the completion of all predefined cycles of chemotherapy was not associated with a higher pCR. The AUC was 0.65 (*p*=0.001) (**Supplemental Figure 1B**). Patients with higher clinical model scores (>-0.5395) were associated with higher pCR (58% *vs.* 29.6%, *p* = 0.001). This model was also validated using clinical data from GSE22358 and GSE130788 cohorts and the AUC were 0.75 (*p*=0.045) and 0.63 (*p*=0.045), respectively. All data from GSE22358, GSE37946, GSE50948, GSE76360 and GSE130788 were pooled together. A total of 268 patients were retrieved, and 116 patients (43.3%) achieved pCR. The predictive value of clinical factors was still stable (AUC = 0.61, *p* = 0.001).

### Cluster Analysis of HER2+ Breast Cancer Patients in Neoadjuvant Setting

Cluster analysis was used for the patients in the training cohort, and two clusters were found. In cluster 1, the pCR rate was 17.17% (34/198), among which most patients were ER^+^ (192/198) or PR^+^ (161/198). In cluster 2, the pCR rate was 53.43% (187/350), and most of patients were ER^-^ (330/350) and PR^-^ (344/350). Groups stratified according to the ER and PR status were: ER^+^PR^+^, ER^+^PR^-^, ER^-^PR^+^, ER^-^PR^-^. The pCR rates were 49.69% for ER^-^PR^-^ (161/324), 66.67% for ER^-^PR^+^ (8/12), 36.84% for ER^+^PR^-^(21/57), and 20% for ER^+^PR^+^ (31/155). All ER^+^PR^+^ patients were in cluster 1, and all ER^-^PR^-^ patients were in cluster 2 (**Supplemental Figure 2**). The ER^-^PR^-^ subtype showed more sensitivity than the ER^+^PR^+^ subtype with wPC (57.64% *vs.* 25.69%), 3wPC (51.16% *vs.* 16.00%), AC-T/P (45.16% *vs.* 9.52%), TAC (25.00% *vs.* 0%). The pCR rates in ER^-^PR- were higher than that in ER^+^PR^+^ breast cancer based on data in the GSE22358 (15/19 *vs.* 0/10, *p <*0.001) and GSE50948 (26/43 *vs.* 1/7, *p*=0.023) cohorts.

**Figure 3:**
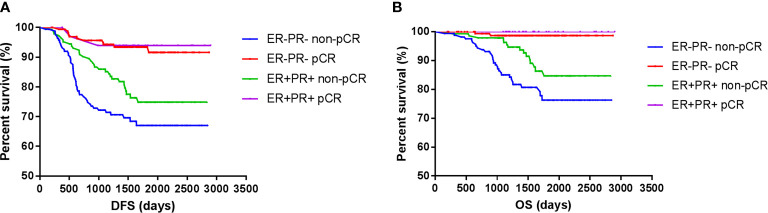
Survival according to pathological complete response and ER/PR. [**(A)** disease free survival; **(B)** over all survival].

However, the DFS and OS in ER^-^PR^-^ patients did not differ from those with ER^+^PR^+^ HER2+ breast cancer patients. Among those who achieved pCR, no survival differences were observed between ER^+^PR^+^ and ER^-^PR^-^ patients, but ER^+^PR^+^ patients showed significant survival benefits than ER^-^PR^-^ patients among those who did not achieve pCR. Whether ER^+^PR^+^ or ER^-^PR^-^ breast cancer patients, achieving pCR could improve survival (**Figure 3**).

### Genetic Characteristics of Patients Who Did Not Achieve pCR

**Figure 4:**
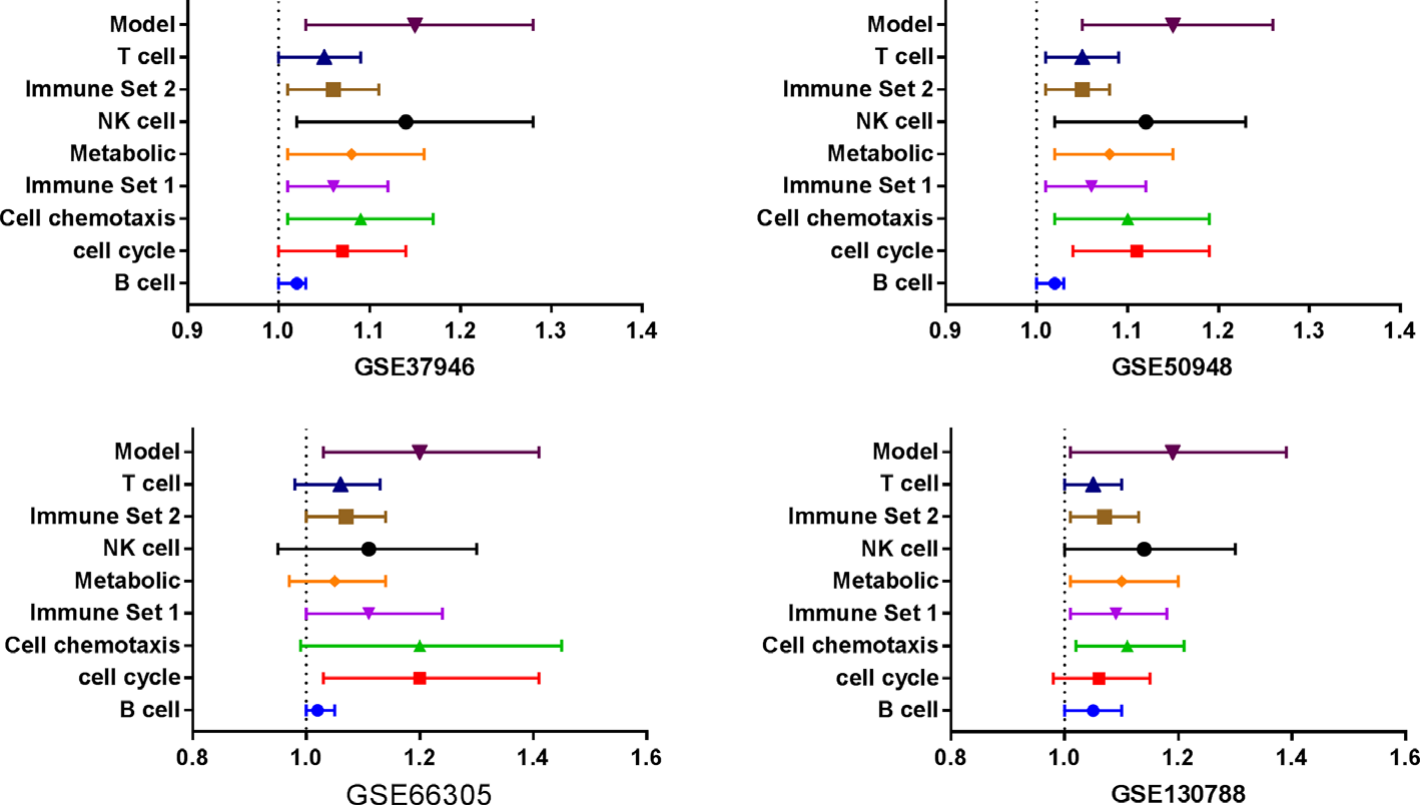
Immune signatures and model between pathological complete response and non- pathological complete response in different datasets.

Three databases (GSE37946, GSE50948, and GSE66305) were chosen as training cohorts, and 74 DEGs with the same trends in at least two databases were retrieved (**Supplemental Table 13**). These genes were T, B, and NK cell-associated genes, chemokines, genes in metabolic and cell proliferation pathways. T, B, NK cell, chemokine, genes in metabolic and cell proliferation pathways, and immune set 2 signatures were built (**Supplemental Table 14**). Low expressions of B cell, chemotaxis, immune set 2 and cell proliferation pathways were significantly correlated with lower probability of achieving pCR in all these datasets, while low expressions of T, NK, metabolic pathways were only correlated with lower probability of achieving pCR in GSE37946 and GSE50948 (**Figure 4** and **Supplemental Table 15**). Using GSE130788 as a validation cohort, low expression levels of B, T, NK cell, chemotaxis, immune set 2, and metabolic pathways were associated with lower probability of achieving pCR (**Supplemental Table 15**).

Seventeen DEGs (GBP1, IGHM, IGKC, IGLC1, CXCL10, CXCL11, CXCL13, SP140, IGLJ3, IGK, UGT2B28, IGLL5, AC128677.4, IGKV1-17, IGKV1-37, IGKV1OR2-108, M24668) were identified, which showed lower expression in non-pCR patients (**Table 2**). These genes participate in the immune response, including B cell activation (IGKC, IGHM, IGLL5, IGLC1), cell chemotaxis (CXCL11, CXCL10, CXCL13), and phagocytosis (IGKV1-17, IGKC, IGLC1). These 17 genes might be classified as B-cell associated genes (IGHM, IGK, IGKC, IGKV1-17, IGKV1-37, IGKV1OR2-108, IGLC1, IGLJ3, IGLL5), T-cell associated signal transduction genes (GBP1, SP140), chemokines (CXCL10, CXCL11, CXCL13), and others (UGT2B28, AC128677.4, M24668). Four clusters could be developed based on these genes, and the higher total score was associated with higher pCR probability. The coefficient was calculated using lasso methods for each classification, and finally a formula was proposed:

**Table 2:** The DEGs across three cohorts (17 genes).

ID	GENE	GSE37946		GSE50948		GSE66305	
		logFC	*p*	logFC	*p*	logFC	*p*
AC128677.4	AC128677.4	0.86	0.01	0.41	0.04	2.01	0.004
3627	CXCL10	0.67	0.02	0.67	0.02	1.72	0.01
6373	CXCL11	0.53	0.03	0.44	0.03	1.80	0.02
10563	CXCL13	1.29	0.01	0.76	0.01	2.60	0.003
2633	GBP1	0.68	0.04	0.43	0.04	1.15	0.02
3507	IGHM	0.90	0.01	0.50	0.01	1.93	0.01
50802	IGK	0.61	0.04	0.49	0.02	1.79	0.02
3514	IGKC	0.73	0.02	0.61	0.01	1.70	0.01
IGKV1-17	IGKV1-17	1.18	0.01	0.85	0.01	3.01	0.0001
IGKV1-37	IGKV1-37	1.05	0.01	0.56	0.01	2.50	0.003
IGKV1OR2-108	IGKV1OR2-108	1.05	0.01	0.49	0.02	2.31	0.002
3537	IGLC1	0.65	0.04	0.52	0.01	1.29	0.04
28831	IGLJ3	0.70	0.01	0.60	0.001	1.64	0.02
100423062	IGLL5	0.59	0.04	0.54	0.005	2.24	0.01
M24668	M24668	0.64	0.04	0.58	0.003	2.08	0.01
11262	SP140	0.50	0.05	0.44	0.02	1.30	0.04
54490	UGT2B28	0.70	0.02	0.84	0.003	2.29	0.046

y2 = (0.6×CXCL13 + 0.2×CXCL10 + 0.2×CXCL11) + (0.10×IGKV1OR2108 + 0.70×IGLJ3 + 0.20×IGKV117) + (0.5×GBP1 + 0.5×SP140) + (0.5×UGT2B28 + 0.5×AC128677.4).

This model showed a high predictive ability for pCR in the GSE37946 (AUC = 0.72, p = 0.008), GSE50948 (AUC = 0.71, *p* = 0.005), and GSE66305 (AUC = 0.90, *p* = 0.004) datasets. Using GSE130788 as a validation cohort, the AUC value was 0.72 (*p* = 0.04).

According to the ROC results, the best cutoff points were calculated. For those whose model score was less than 10, the pCR probability ranged from 0% to 28.6%, and for those whose model score was more than 20, the pCR probability ranged from 57.1% to 80%. After adjusting for ER and PR status, this model was still associated with pCR (**Supplemental Table 16**).

### Combination of Clinical and Genetic Variables for Predicting pCR in HER2+ Breast Cancer

Each gene in genetic models was standardized from 0 to 10. There were four parts for genetic model, so the total score ranged from 0 to 40. There were two parts for clinical model (pathological and treatment factors), and the clinical models were standardized from 0 to 20. Multivariate logistic analyses were conducted to analyze the coefficient for clinical and genetic model (y1 and y2) in GSE22358, GSE37946, GSE50948 and GSE130788. The relative ratio (RR) between the coefficient for y2 and y1 ranged from 1 to 13. The best RR was 2, so a formula which combined clinical and genetic models was proposed:

y3=y1*20/3+y2*2/3.

The AUC values for the combination modes were 0.79 (*p* = 0.014) for GSE22358, 0.71 (*p* = 0.014) for GSE37946, 0.64 (*p* = 0.012) for GSE50948, 0.72 (*p* = 0.04) for GSE130788.

### ER^-^PR^-^ Might Be Not Associated With Higher Immune Cell Infiltration Than ER^+^PR^+^ Breast Cancer

We further used all data in the GSE50948 and GSE130788 cohorts to analyze differences in the signatures and models between ER^+^PR^+^ and ER^-^PR^-^ breast cancer patients (**Supplemental Tables 17**, **18**). Based on these data, ER^-^PR^-^ patients were associated with higher scores in B, T, NK cell, chemotaxis, immune set 2, and metabolic signatures. However, in the GSE37946 and GSE58984 datasets, no significant differences were observed between ER^+^PR^+^ and ER^-^PR^-^ breast cancer patients (**Supplemental Tables 19**, **20**), which suggested HR status and immune signatures were independent predictive factors for pCR. Analyzing the interaction across ER status, PR status, and the predictive model, no significant differences were found. Intratumoral lymphocytic infiltration and stromal lymphocytic infiltration was associated with increased scores in B, T, NK cell, chemotaxis, immune set 2, and metabolic signatures, as well as the model score in GSE58984 (**Table 3**).

**Table 3:** The signatures in GSE58984 between different intratumoral lymphocytic infiltration (TIL) and stromal lymphocytic infiltration (SLI) statuses.

Signature	TIL +	TIL -	p value	SLI +	SLI -	p value
B cell	110.45 ± 34.72	76.5 ± 32.25	<0.001	118.95 ± 29.23	70.98 ± 28.39	<0.001
cell cycle	33.91 ± 8.18	30.48 ± 6.81	0.03	35.41 ± 7.78	29.51 ± 6.42	0.0001
Cell chemotaxis	34.38 ± 8.16	24.23 ± 8.89	<0.001	35.31 ± 7.25	23.63 ± 8.64	<0.001
Immune Set 1	42.4 ± 12.37	32.79 ± 9.44	<0.001	44.15 ± 11.04	31.65 ± 9.14	<0.001
Metabolic	32.77 ± 6.76	31.17 ± 8.83	0.35	33.76 ± 7.44	30.53 ± 8.28	0.06
NK cell	19.33 ± 6.42	13.88 ± 5.45	<0.001	20.47 ± 5.4	13.14 ± 5.27	<0.001
Immune Set 2	64.75 ± 13.33	61.31 ± 10.39	0.17	67.82 ± 12.94	59.32 ± 9.49	0.0004
T cell	46.58 ± 14.61	33.97 ± 11.33	<0.001	48.78 ± 12.51	32.54 ± 11.09	<0.001
Model	22.76 ± 5.52	15.85 ± 5.79	<0.001	23.92 ± 4.67	15.1 ± 5.2	<0.001

## Discussion

Our study developed a clinical model for pCR in HER2+ breast cancer patients receiving neoadjuvant chemotherapy and trastuzumab using real world data, which consisted of preoperative ER status, PR status, HER2+ expression, chemotherapy regimens, and completion of all predefined cycles of chemotherapy. Although this clinical model integrated more clinical variables and was validated by an independent cohort and other published cohorts, this model was limited in the ability for the prediction of pCR. We also explored the predictive values of genetic data on pCR, and developed genetic models for those who received trastuzumab. This genetic model has a wider applicability and allows greater generalization.

Years ago, women with HER2+ breast cancer were associated with worse prognosis (15). But trastuzumab drastically changed outcomes and substantial evidence has now shown that trastuzumab treatment could improve patient survival. A Cochrane review study based on 8 studies (11,991 patients) showed that trastuzumab-based regimens significantly improved OS (HR 0.66, 95% CI 0.57-0.77) and DFS (HR 0.60, 95% CI 0.50-0.71) (16). Tang et al. showed that 12 months of trastuzumab significantly reduced overall mortality (HR 0.71, 95% CI 0.62-0.81) as compared to those who received <12 months of therapy (17). All these data were about those who received adjuvant trastuzumab. Our study confirmed adequate (12 months) trastuzumab therapy could prolong the survival of HER2+ breast cancer patients in terms of DFS (HR 0.49, 95% CI 0.29-0.85) and OS (HR 0.30, 95% CI 0.15-0.60) in the neoadjuvant setting.

Substantial evidence from clinical trials confirmed that achieving pCR was correlated with long-term survival benefits in HER2+ breast cancer (18). Cortazar et al. showed that pCR was associated with long-term outcome (event-free survival (EFS): HR 0.39, 95% CI 0.31-0.50; OS: 0.34, 0.24-0.47) for HER2+ breast cancer patients (8). Our retrospective cohort of 600 HER2+ breast cancer cases in the neoadjuvant treatment setting confirmed pCR was associated with better survival irrespective of the patients’ clinical and pathological characteristics, and could be used to predict these patients’ prognosis.

For HER2+ breast cancer patients in neoadjuvant setting, the standard regimen to date was chemotherapy and trastuzumab (AC-TH, AC-PH, or TCbH), in which the pCR rates of 50% or more can be achieved (15, 19–21). TCbH has often been preferred by clinicians due to its lower cardiotoxicity profile, in which weekly regimens are much more effective than the three-week schedule (69% *vs.* 41%; p = 0.03) (15, 21). In our study, we separately analyzed the influence of neoadjuvant chemotherapy, trastuzumab use and completion of chemotherapy cycles on pCR, and found wPC, trastuzumab, and a sufficient number of cycles completed were associated with higher pCR rates. The weekly PC has higher pCR rate than the three-week schedule (46.70% *vs.* 35.53%, *p*=0.08). Interesting, the AC-T/P has a similar pCR rate as 3wPC (33.85% *vs.* 35.53%, *p*=0.84), which was lower than wPC (46.70% *vs.* 33.85%, *p*=0.06). The TAC regimen had the lowest pCR rate, which may have been due to the impossibility of trastuzumab use, which decreased the pCR rate. Our study showed that trastuzumab use could at least double the pCR rates (OR 3.47, 95% CI 1.93-6.22). Although the pCR rate achieved 39.8%, most patients did not achieve pCR. Thus, the identification of patients not sensitive to treatment is critical for improving their prognosis. Based on our study, ER and PR positivity, low Ki-67 and HER2 were associated with lower pCR. For those patients, how to improve the efficacy of NACT warrants further research.

Several nomograms have been developed to predict pCR for breast cancer patients, however, most considered HER2-negative patients alone (22). Using these to predict the pCR for HER2+ breast cancer patients might not be suitable. There were lots of studies that aimed to evaluate the predictive values of some clinical and pathological factors, such as hormone receptor (23, 24), trastuzumab use (24). The nomograms based on these factors were proposed (25, 26). The first nomogram to predict pCR for HER2+ breast cancer patients treatment with trastuzumab was developed by Jankowski et al. (25). This model was based on 101 patients and could be applied to those with trastuzumab without considering chemotherapy regimens and cycles. Thus, this model could not be generalized to conditions in which patients did not receive trastuzmab, had received different chemotherapy regimens, or to stage T4 patients. Fujii et al. developed a nomogram which consisted of ER, PR, HER2 FISH ratio, inflammatory breast cancer and neoadjuvant systemic therapy regimen based on 793 patients (26). The strength of this model is that it could be used to predict pCR for those who received pertuzumab, but this model did not consider different chemotherapy regimens. However, available models could not fully utilize available clinical and pathological information. Our model included ER, PR, and HER2 status, trastuzumab use, neoadjuvant chemotherapy, and chemotherapy cycles, which has a wider applicability and allows greater generalization. Our validation cohort and testing in the published cohort confirmed the stability of this model.

There were lots of studies that aimed to identify the biomarkers with the best association with pCR in response to trastuzumab (27–30), but there are currently no conclusive biomarkers for patient response to trastuzumab (31). Tanioka et al. developed a multi-dimensional genomic analysis to integrate DNA mutations, DNA copy number aberrations, and RNA transcriptional expression with clinical variables using prospectively collected frozen tissue samples from a Phase III trial to predict pCR (28). This model reported higher predictive values, but is difficult to apply into routine clinical practice. Fernandez-Martinez et al. (32) found a total of 215 genomic variables were significantly associated with pCR. Among these genetic models, cases that achieved a pCR had evidence of an activated immune response, including g T-Cell, B-Cell, and inflammatory signatures (33). However, these models were not easy to apply into routine clinical practice and need to be confirmed by other cohorts. Thus we analyzed the available RNA sequence databases in order to develop a genetic model which could be confirmed by other published cohorts. Seventy-four genes showed same trends in at least two databases, and 17 genes were higher in pCR patients in all three databases. These 17 genes could be classified as T, B, NK cell, cell proliferation, cell metabolism, and chemotaxis signatures. Across four different datasets, these immune signatures were higher in pCR patients than in non-pCR. The 17 DEGs identified were associated with the immune response in breast cancer patients, including B cell activation (IGKC, IGHM, IGLL5, IGLC1), cell chemotaxis (CXCL11, CXCL10, CXCL13), phagocytosis (IGKV1-17, IGKC, IGLC1). CXCL13, which might suppress regulatory T (Treg)-mediated immune response and activation of adaptive antitumor humoral responses (34) and thus, might be associated with higher pCR in neoadjuvant setting (35). The CXC subfamily chemokines (CXCL10, CXCL11, and CXCL13) might induce the migration mainly of T cells and B cells (36). IGHM and IGLJ3, B cell-specific immunoglobulin, have been reported as adaptive immunity effector genes (37). Studies have shown that higher CXCL13 and SP140 expression were associated with increased recurrence free survival (RFS) for those receiving adjuvant trastuzumab (38). However, the roles of these genes in trastuzumab resistance have not been studied. In our study, we developed a predictive model that also consisted of genes related to T, B cell and cell chemokines. Our model could predict the pCR and multivariate logistic analysis showed this model could predict pCR independently.

In our study, we identified HR status and immune cell infiltrations as two independent predictive factors for pCR in HER2+ breast cancer. Plenty of evidence has shown that ER is an independent predictive factor in breast cancer (28, 39–41) and inhibition of the ER might enhance responses to trastuzumab in HER2 positive breast cancer cells (40). However, clinical study did not confirm the additional benefits by concurrent targeting of ER and HER2. NSABP B-52 study randomly assigned 315 patients to receive neoadjuvant therapy consisting of docetaxel, carboplatin, trastuzumab, and pertuzumab with or without estrogen deprivation therapy. This study failed to show superior benefits in pCR (40.9% *vs.* 46.1%, p = 0.36) (42). Several immune-cell signatures have been reported, which were highly associated with pCR, but the models were not adequately cross-validated by independent cohorts or by other researchers (43, 44). Our model was validated in four datasets and showed stable predictive ability. Nonetheless, this model was not confirmed by real-time quantitative PCR validation. But we have an independent cohort which was used to test the model. Meanwhile, we found that the immune signatures and model scores were different for ER^+^PR^+^ and ER^-^PR^-^ patients, which might suggest that HR status might be associated with immune cell infiltrations. We then analyzed GSE58984 and GSE37946 in this regard, this was not well confirmed. Likewise, no interaction between HR and immune signatures for pCR were found. This might be due to different lymphocytic infiltrations, whose levels were associated with a higher probability of pCR and immune signature scores. Continuous stromal-tumor-infiltrating lymphocytes (TIL)s (OR 1.03, 95% CI 1.02-1.05, *p*<0.001) and intratumoral-TILs (OR 1.09, 95% CI 1.04-1.15, *p*<0.001) were significantly associated with pCR (45). In our study, higher TILs were associated with higher immune signatures and model scores, which further validated the stability of our model. T and B cell infiltrations have often been reported in previous studies (43–45). In our study we found that NK cell activation might be associated with pCR. The NK cell signatures were higher in pCR patients across the three datasets tested. Recruitment of NK cells and subsequent induction of antibody-dependent cell-mediated cytotoxicity (ADCC) contributed to this beneficial effect (46).

For those who were unlikely to achieve pCR with standard treatment, how to improve the possibility of pCR was still required further research. NeoSphere study showed patients given pertuzumab and trastuzumab plus docetaxel had a highest pCR (49/107) compared with those given trastuzumab plus docetaxel (31/107, p=0.014) (30). PEONY study showed the pCR rates were 39.3% (86 of 219) in the pertuzumab and trastuzumab group and 21.8% (24 of 110) in the trastuzumab group (p = 0.001) (47). This study confirmed the additional benefits in pCR when adding pertuzumab for ER^-^PR^-^ breast cancer patients, but not for ER^+^ and/or PR^+^ breast cancer patients. CALGB 40601 study (43) randomly assigned 305 patients to paclitaxel, trastuzumab, lapatinib (THL), paclitaxel plus trastuzumab (TH), or paclitaxel plus lapatinib (TL). The additional use of laptinib did not increase the pCR rate (56% *vs.* 46%, p = 0.13). Subgroup analysis confirmed similar results in HR^+^ breast cancer (41% *vs.* 41%), but THL was associated with higher pCR rates than TH (79% *vs.* 54%, p = 0.01). TEAL study (48) showed the proportion of patients with RCB 0 or I in the T-DM1, lapatinib, nab-paclitaxel group was higher than that in trastuzumab, pertuzumab and paclitaxel group (100% *vs.* 62.5%, *p* = 0.0035). ER^-^ patients in both groups achieved RCB-0 or RCB-I, but all ER^+^ patients in the experimental arm achieved RCB 0-I *versus* 25% in the standard arm (*p* = 0.0035). In total, for ER^-^ breast cancer patients, adding pertuzumab or lapatinib might improve the pCR rates, but for ER^+^ breast cancer, this might not work. For ER+ breast cancer patients, how to improve the pCR rates deserved further research. Although bispecific antibody (BsAb) simultaneously targeting both PD1 or PD-L1 and HER2 inhibited tumor growth (49–51), no clinical studies in neoadjuvant setting were conducted. Whether adding immunotherapy to enhance immune recognition of tumors with low immune markers deserved further research. Even for those who did not achieve pCR, adjuvant T-DM1 might reduce the risk of recurrence as compared to trastuzumab (52).

## Strength and Limitations

Our study was the largest retrospective cohort in Chinese HER2+ breast cancer patients in the neoadjuvant setting. We analyzed clinical factors that might affect the pCR and developed a model that could be used to predict pCR. Although some of the factors have already been used by other groups to build prediction models, our study was the first one that assessed and incorporated all relevant important clinical factors. All clinical variables were analyzed. In addition, we developed a genetic model to predict pCR, which showed stability across different databases. Nonetheless, our study also had its limitations. First, all data was collected retrospectively with the inherent biases deriving from data collection, although three co-authors verified the data. Our study was based on real-world data, and many unevaluated variables that might have affected the outcomes. For example, there was a high proportion (53%) of patients who did not complete all predefined NACT cycles and whose pCR rate was 33.96%. Second, although the genetic model was validated by other databases, its real value in clinical practice should be determined in future studies, alongside its further confirmation by RT-QPCR, *in vitro* or *in vivo* experiments. Third, this study developed clinical and genetic models for HER2+ breast cancer patients who received neoadjuvant chemotherapy and trastuzumab, so whether these models could predict pCR for those who received pertuzumab or tyrosine kinase inhibitors deserved further research. Meanwhile, the size of the training and validation cohort is small. The validation cohort is from Chinese population and the model needs to be tested with other ethnicity cohorts to have global use.

## Conclusion

We determined that pCR was an independent prognostic factor for DFS and OS. Further, ER, PR, and HER2 status were associated with pCR, and use of wPC, and trastuzumab with a sufficient number of cycles completed could improve the possibility of achieving a pCR. The model we developed could be used for the prediction of pCR in HER2+ breast cancer patients. Genetic data including RNA expression evaluation might be helpful to guide clinical treatment and could be used for the prediction of clinical outcomes. However, both clinical and genetic models should be verified in other clinical contexts.

## Data Availability Statement

The original contributions presented in the study are included in the article/**Supplementary Material**. Further inquiries can be directed to the corresponding author.

## Ethics Statement

The studies involving human participants were reviewed and approved by Ethics Committee in Fudan University Shanghai Cancer Center. Written informed consent for participation was not required for this study in accordance with the national legislation and the institutional requirements.

## Author Contributions 

Study design: LL and JioW. Data collection: LL, MC, SZ, and HL. Data analysis: LL and JioW. Manuscript writing: LL, MC, SZ, WC, BX, QZ, JiaW, JH, HL, and JioW. All authors contributed to the article and approved the submitted version.

## Funding

This study was funded by the Academic Leaders of Shanghai Science and Technology Commission (18XD1401300), the Open Fund of the Key Laboratory of Evidence Based Medicine and Knowledge Translation of Gansu Province (GSKL-EBM&KT-201902), and Youth Program of National Natural Science Foundation of China (82002797).

## Conflict of Interest

The authors declare that the research was conducted in the absence of any commercial or financial relationships that could be construed as a potential conflict of interest.
