# Supplementary material for: Clinical and Genetic Predictive Models for the Prediction of Pathological Complete Response to Optimize the Effectiveness for Trastuzumab Based Chemotherapy
Source: Front Oncol. 2021 Jul 15;11:592393. doi: 10.3389/fonc.2021.592393 (PMC8319743; doi:10.3389/fonc.2021.592393)
Supplement: Supplementary file 1 [file DataSheet_1.doc]

Supplemental table 1 characteristics of published cohorts

| GSE ID | Regimens | Chemotherapy | Trastuzumab durations | pCR/RD |
| --- | --- | --- | --- | --- |
| 37946 | anthracyclines based | fluorouracil/epirubicin or driamycin/cyclophosphamide-taxol | - | 27/22 |
| 50948 | anthracyclines based | doxorubicin/paclitaxel followed by cyclophosphamide/methotrexate/fluorouracil | 1 year of treatment with trastuzumab given as neoadjuvant and adjuvant treatment | 31/32 |
| 66305 | anthracyclines based | weekly paclitaxel for 12 weeks followed by fluorouracil, epirubicin, and cyclophosphamide | trastuzumab (4-mg/kg loading dose followed by 2 mg/kg weekly for 26 weeks) | 8/16 |
| 130788 | anthracyclines free | docetaxel and carboplatin with trastuzumab | Patients receive docetaxel IV, carboplatin IV, and trastuzumab IV over 30 minutes on day 1 in course 2-7. Courses repeat every 21 days in the absence of disease progression or unacceptable toxicity. | 15/16 |

Supplemental table 2 characteristics of the independent validation cohort in FUSCC

| Variables |  | No. | Non- pCR | pCR | X^2^ | p |
| --- | --- | --- | --- | --- | --- | --- |
| T | cT1 | 15 | 6 | 9 | 6.73 | 0.15 |
|  | cT2 | 99 | 52 | 47 |  |  |
|  | cT3 | 39 | 23 | 16 |  |  |
|  | cT4 | 10 | 8 | 2 |  |  |
| N | cN0 | 15 | 6 | 9 | 2.56 | 0.47 |
|  | cN1 | 125 | 68 | 57 |  |  |
|  | cN2 | 5 | 2 | 3 |  |  |
|  | cN3 | 20 | 13 | 7 |  |  |
| ER | negative | 100 | 44 | 56 | 10.09 | 0.001 |
|  | positive | 65 | 45 | 20 |  |  |
| PR | negative | 118 | 56 | 62 | 7.01 | 0.008 |
|  | positive | 47 | 33 | 14 |  |  |
| HER2 | 1+ | 5 | 4 | 1 | 9.89 | 0.007 |
|  | 2+ | 38 | 28 | 10 |  |  |
|  | 3+ | 122 | 57 | 65 |  |  |
| Ki-67 | <20% | 12 | 10 | 2 | 4.50 | 0.034 |
|  | ≥20% | 153 | 79 | 74 |  |  |
| Chemotherapy | wPC | 114 | 61 | 53 | 10.97 | 0.027 |
|  | 3wPC/TC | 18 | 13 | 5 |  |  |
|  | AC-T/P | 25 | 8 | 17 |  |  |
|  | TAC(PAC, PEC, TEC) | 1 | 1 | 0 |  |  |
|  | Other | 7 | 6 | 1 |  |  |
| Trastuzumab use | no | 11 | 10 | 1 | 6.29 | 0.01 |
|  | yes | 154 | 80 | 74 |  |  |
| All predefined cycles | no | 60 | 34 | 26 | 0.28 | 0.60 |
|  | yes | 105 | 55 | 50 |  |  |

Supplemental table 3: Cox proportional hazards regression analysis of DFS for the training cohort

| Variables | Categories | No. | Univariate COX analysis | | Multivariate COX analysis | |
| --- | --- | --- | --- | --- | --- | --- |
|  |  |  | HR (95%CI) | *p* | HR (95%CI) | *p* |
| Clinical T stage | cT1 | 48 | Ref |  | Ref |  |
|  | cT2 | 330 | 1.21(0.52-2.82) | 0.66 | 0.74(0.28-1.99) | 0.55 |
|  | cT3 | 144 | 2.05(0.86-4.87) | 0.11 | 1.9(0.7-5.15) | 0.21 |
|  | cT4 | 67 | 2.82(1.14-6.98) | 0.03 | 1.4(0.48-4.14) | 0.54 |
| Clinical N stage | cN0 | 150 | Ref |  | Ref |  |
|  | cN1 | 346 | 1.33(0.81-2.18) | 0.26 | 0.91(0.52-1.6) | 0.74 |
|  | cN2 | 33 | 1.7(0.76-3.85) | 0.2 | 0.82(0.33-2.04) | 0.67 |
|  | cN3 | 69 | 2.25(1.22-4.16) | 0.01 | 1.62(0.78-3.39) | 0.2 |
| Age | <35 | 58 | Ref |  | Ref |  |
|  | 35-65 | 511 | 0.75(0.43-1.32) | 0.32 | 0.76(0.39-1.5) | 0.43 |
|  | >65 | 31 | 0.88(0.34-2.3) | 0.8 | 1.15(0.38-3.45) | 0.81 |
| BMI | Obesity vs. normal | 406/194 | 1.04(0.7-1.54) | 0.85 | 0.75(0.47-1.2) | 0.23 |
| Menopausal status | yes vs. no | 241/359 | 0.98(0.68-1.43) | 0.94 | 1.02(0.66-1.58) | 0.93 |
| Pathological T stage | pT0 | 232 | Ref |  | Ref |  |
|  | pTis | 59 | 1.21(0.49-3) | 0.68 | 1.68(0.64-4.43) | 0.29 |
|  | pT1 | 205 | 2.45(1.46-4.13) | 0.001 | 2.35(1.24-4.42) | 0.01 |
|  | pT2 | 95 | 5.78(3.4-9.84) | <0.001 | 4.56(2.24-9.29) | <0.001 |
|  | pT3/4 | 9 | 4.57(1.36-15.31) | 0.01 | 3.47(0.73-16.6) | 0.12 |
| Pathological N stage | pN0 | 388 | Ref |  | Ref |  |
|  | pN1 | 118 | 2.38(1.45-3.9) | <0.001 | 1.64(0.87-3.08) | 0.13 |
|  | pN2 | 56 | 5.8(3.55-9.48) | <0.001 | 3.91(1.98-7.71) | <0.001 |
|  | pN3 | 29 | 9.11(5.15-16.13) | <0.001 | 7.85(3.63-16.97) | <0.001 |
| Postoperative pathological type(#) | No invasive tumor | 232 | Ref |  | Ref |  |
|  | DCIS | 59 | 1.21(0.49-2.99) | 0.68 | 1.66(0.63-4.37) | 0.307 |
|  | IDC | 245 | 3.39(2.08-5.53) | <0.001 | 2.82(1.51-5.28) | 0.001 |
|  | IDC+DCIS | 64 | 3.47(1.83-6.57) | <0.001 | 2.89(1.34-6.25) | 0.007 |
| pCR* | yes vs. no | 239/361 | 0.23(0.13-0.38) | <0.001 | 0.37(0.16-0.88) | 0.02 |
| ER | Positive vs. negative | 346/254 | 1.03(0.71-1.5) | 0.87 | 1.8(0.82-3.95) | 0.14 |
| PR | Positive vs. negative | 404/196 | 0.92(0.62-1.37) | 0.67 | 0.72(0.36-1.45) | 0.36 |
| Her2 | 3+ | 528 | Ref |  | Ref |  |
|  | 2+ | 62 | 1.72(0.54-5.43) | 0.36 | 1.41(0.41-4.87) | 0.58 |
|  | 1+ | 10 | 1.31(0.75-2.3) | 0.35 | 0.76(0.38-1.53) | 0.44 |
| Ki67 | ≥20% vs. <20% | 545/55 | 1.04(0.54-1.98) | 0.92 | 0.99(0.45-2.2) | 0.99 |
| Breast Surgery | Mastectomy vs. BCS | 525/75 | 0.69(0.37-1.29) | 0.24 | 1.51(0.73-3.12) | 0.26 |
| Axillary surgery | ALND | 479 | Ref |  | Ref |  |
|  | SLNB | 48 | 0.42(0.15-1.14) | 0.09 | 0.87(0.29-2.66) | 0.81 |
|  | SLNB-ALND | 70 | 0.98(0.56-1.73) | 0.96 | 1.03(0.54-1.97) | 0.94 |
| Trastuzumab | >12 months vs. <12months/no | 517/83 | 0.43(0.27-0.68) | <0.001 | 0.49(0.29-0.85) | 0.01 |
| Endocrine therapy | yes vs. no | 233/367 | 0.51 (0.23-1.14) | 0.10 | 0.30(0.10-0.89) | 0.03 |
| Radiotherapy | yes vs. no | 461/139 | 0.92(0.62-1.36) | 0.67 | 1.2(0.65-2.19) | 0.56 |
| Adjuvant chemotherapy | yes vs. no | 349/251 | 1.37(0.92-2.04) | 0.13 | 1.08(0.68-1.73) | 0.74 |

Supplemental table 4: Cox proportional hazards regression analysis of OS for the training cohort

| Variables | Categories | No. | Univariate COX analysis | | Multivariate COX analysis | |
| --- | --- | --- | --- | --- | --- | --- |
|  |  |  | HR (95%CI) | *p* | HR (95%CI) | *p* |
| Clinical T stage | cT1 | 48 | Ref |  | Ref |  |
|  | cT2 | 330 | 1.31(0.39-4.33) | 0.66 | 1.45(0.27-7.63) | 0.66 |
|  | cT3 | 144 | 2.28(0.68-7.72) | 0.18 | 5.06(0.91-28.2) | 0.07 |
|  | cT4 | 67 | 3.75(1.07-13.16) | 0.04 | 5.14(0.86-30.56) | 0.07 |
| Clinical N stage | cN0 | 150 | Ref |  | Ref |  |
|  | cN1 | 346 | 1.83(0.84-3.97) | 0.13 | 1.79(0.72-4.44) | 0.21 |
|  | cN2 | 33 | 2.75(0.9-8.4) | 0.08 | 1.97(0.55-7.14) | 0.3 |
|  | cN3 | 69 | 3.98(1.67-9.49) | 0.002 | 5.03(1.61-15.66) | 0.005 |
| Age | <35 | 58 | Ref |  | Ref |  |
|  | 35-65 | 511 | 1(0.43-2.33) | 1 | 1.27(0.42-3.82) | 0.67 |
|  | >65 | 31 | 2.44(0.79-7.58) | 0.12 | 2.83(0.61-13.17) | 0.19 |
| BMI | BMI | 406/194 | 1.03(0.6-1.76) | 0.92 | 0.74(0.37-1.49) | 0.4 |
| Menopausal status | yes vs. no | 241/359 | 1.29(0.76-2.18) | 0.34 | 0.99(0.51-1.89) | 0.96 |
| Pathological T stage | pT0 | 232 | Ref |  | Ref |  |
|  | pTis | 59 | 0.52(0.07-4.19) | 0.54 | 0.86(0.09-8.02) | 0.9 |
|  | pT1 | 205 | 3.59(1.62-7.96) | 0.00165 | 3.09(1.11-8.64) | 0.03 |
|  | pT2 | 95 | 8.69(3.9-19.35) | <0.001 | 5(1.7-14.7) | 0.003 |
|  | pT3/4 | 9 | 6.6(1.4-31.1) | 0.02 | 3.01(0.3-30.03) | 0.35 |
| Pathological N stage | pN0 | 388 | Ref |  | Ref |  |
|  | pN1 | 118 | 3.06(1.53-6.13) | 0.002 | 2.3(0.9-5.89) | 0.08 |
|  | pN2 | 56 | 5.05(2.36-10.79) | <0.001 | 2.73(0.94-7.9) | 0.06 |
|  | pN3 | 29 | 15.26(7.7-30.22) | <0.001 | 20.91(8.06-54.27) | <0.001 |
| Postoperative pathological type | No invasive tumor | 232 | Ref |  | Ref |  |
|  | DCIS | 59 | 0.52(0.07-4.19) | 0.54 | 0.8(0.09-7.54) | 0.85 |
|  | IDC | 245 | 5.02(2.35-10.71) | 0.00003 | 3.75(1.39-10.17) | 0.009 |
|  | IDC+DCIS | 64 | 5.37(2.12-13.63) | 0.0004 | 3.4(1-11.51) | 0.05 |
| pCR* | pCR* | 239/361 | 0.08(0.02-0.24) | <0.001 | 0.09(0.03-0.3) | <0.001 |
| ER | ER | 346/254 | 1.01(0.6-1.68) | 0.98 | 5.76(2.09-15.92) | 0.001 |
| PR | PR | 404/196 | 0.69(0.39-1.22) | 0.2 | 0.32(0.11-0.94) | 0.04 |
| HER2 | her2 3+ | 528 | Ref |  | Ref |  |
|  | her2 2+ | 62 | 2.01(0.49-8.24) | 0.34 | 2.92(0.55-15.54) | 0.21 |
|  | her2 1+ | 10 | 1.22(0.55-2.69) | 0.62 | 0.5(0.15-1.63) | 0.25 |
| Ki67 | ≥20% vs. <20% | 545/55 | 0.75(0.34-1.64) | 0.47 | 0.42(0.15-1.18) | 0.1 |
| Breast Surgery | Mastectomy vs. BCS | 525/75 | 0.11(0.02-0.78) | 0.03 | 0.21(0.02-1.96) | 0.17 |
| Axillary surgery | ALND | 479 | Ref |  | Ref |  |
|  | SLNB | 48 | 0(0-4.47E+264) | 0.97 | 0(0-1.05E+252) | 0.97 |
|  | SLNB-ALND | 70 | 0.63(0.25-1.58) | 0.33 | 1.11(0.38-3.19) | 0.85 |
| Trastuzumab | yes vs. no | 517/83 | 0.24(0.14-0.42) | <0.001 | 0.30(0.15-0.60) | 0.001 |
| Endocrine therapy | yes vs. no | 233/367 | 0.34(0.11-1.03) | 0.06 | 0.10(0.02-0.49) | 0.004 |
| Radiotherapy | yes vs. no | 461/139 | 0.63(0.34-1.18) | 0.15 | 0.57(0.26-1.27) | 0.17 |
| Adjuvant chemotherapy | yes vs. no | 349/251 | 1.72(0.98-3.02) | 0.06 | 1.87(0.89-3.95) | 0.1 |

| All variables were taken account into the multivariable Cox logistic analysis, including clinical T, N, age, BMI, menopausal status, preoperative ER, PR, HER2+, Ki67, surgery, axillary surgery, pT, pN, radiotherapy, endocrine therapy, adjuvant chemotherapy and trastuzumab. |
| --- |
| # pathological types were adjusted for clinical T, N, age, BMI, menopausal status, preoperative ER, PR, HER2+, Ki67, surgery, axillary surgery, pN, radiotherapy, endocrine therapy, adjuvant chemotherapy and trastuzumab |
| *pCR was adjusted for clinical T, N, age, BMI, menopausal status, preoperative ER, PR, HER2+, Ki67, surgery, axillary surgery, radiotherapy, endocrine therapy, adjuvant chemotherapy and trastuzumab |

Supplemental table 5: Cox proportional hazards regression analysis of DFS for the training cohort among those who received neoadjuvant trastuzumab

| Variables | Categories | Univariate COX analysis | | Multivariate COX analysis | |
| --- | --- | --- | --- | --- | --- |
|  |  | HR (95%CI) | *p* | HR (95%CI) | *p* |
| Clinical T stage | cT1 | Ref |  | Ref |  |
|  | cT2 | 1.11(0.44-2.83) | 0.82 |  |  |
|  | cT3 | 2.11(0.82-5.42) | 0.12 |  |  |
|  | cT4 | 2.54(0.93-6.94) | 0.07 |  |  |
| Clinical N stage | cN0 | Ref |  | Ref |  |
|  | cN1 | 1.35(0.77-2.37) | 0.30 | 1(0.55-1.82) | 0.99 |
|  | cN2 | 1.53(0.6-3.92) | 0.37 | 1.09(0.4-2.95) | 0.86 |
|  | cN3 | 2.48(1.25-4.9) | 0.01 | 1.11(0.51-2.43) | 0.79 |
| Age | <35 | Ref |  | Ref |  |
|  | 35-65 | 0.69(0.38-1.28) | 0.24 |  |  |
|  | >65 | 0.96(0.36-2.57) | 0.94 |  |  |
| BMI | Obesity vs. normal | 0.96(0.61-1.5) | 0.84 |  |  |
| Menopausal status | yes vs. no | 0.94(0.62-1.42) | 0.76 |  |  |
| Pathological T stage | pT0 | Ref |  | Ref |  |
|  | pTis | 1.44(0.57-3.65) | 0.44 | 1.38(0.54-3.55) | 0.51 |
|  | pT1 | 2.58(1.43-4.62) | 0.001 | 1.98(1.05-3.74) | 0.03 |
|  | pT2 | 6.82(3.77-12.34) | 0.001 | 4.01(2.01-7.99) | 0.001 |
|  | pT3/4 | 4.24(0.98-18.37) | 0.05 | 1.84(0.39-8.73) | 0.44 |
| Pathological N stage | pN0 | Ref |  | Ref |  |
|  | pN1 | 2.22(1.28-3.84) | 0.004 | 1.45(0.79-2.64) | 0.23 |
|  | pN2 | 5.99(3.51-10.22) | 0.001 | 3.58(1.9-6.73) | 0.001 |
|  | pN3 | 12.01(6.08-23.73) | 0.001 | 6.8(2.97-15.6) | 0.001 |
| Postoperative pathological type(#) | No invasive tumor | Ref |  | Ref |  |
|  | DCIS | 1.44(0.57-3.65) | 0.44 | 1.45(0.55-3.83) | 0.45 |
|  | IDC | 3.62(2.09-6.26) | 0.001 | 2.75(1.45-5.21) | 0.001 |
|  | IDC+DCIS | 4(2-8.01) | 0.001 | 3.13(1.46-6.7) | 0.001 |
| pCR* | yes vs. no | 0.24(0.14-0.42) | 0.001 | 0.22(0.13-0.40) | 0.0001 |
| ER | Positive vs. negative | 1.08(0.71-1.64) | 0.71 |  |  |
| PR | Positive vs. negative | 1.02(0.66-1.59) | 0.92 |  |  |
| Her2 | 3+ | Ref |  | Ref |  |
|  | 2+ | 1.51(0.83-2.72) | 0.17 | 0.88(0.48-1.63) | 0.69 |
|  | 1+ | 3.7(1.17-11.76) | 0.03 | 3.19(0.96-10.64) | 0.06 |
| Ki67 | ≥20% vs. <20% | 0.94(0.45-1.94) | 0.86 |  |  |
| Breast Surgery | Mastectomy vs. BCS | 0.76(0.41-1.43) | 0.40 |  |  |
| Axillary surgery | ALND | Ref |  | Ref |  |
|  | SLNB | 0.46(0.17-1.26) | 0.13 |  |  |
|  | SLNB-ALND | 1.01(0.56-1.82) | 0.98 |  |  |
| Endocrine therapy | yes vs. no | 0.9(0.59-1.37) | 0.62 |  |  |
| Radiotherapy | yes vs. no | 0.94(0.56-1.56) | 0.80 |  |  |
| Adjuvant chemotherapy | yes vs. no | 1.39(0.9-2.16) | 0.14 |  |  |

Supplemental table 6: Cox proportional hazards regression analysis of OS for the training cohort among those who received neoadjuvant trastuzumab

| Variables | Categories | Univariate COX analysis | | Multivariate COX analysis | |
| --- | --- | --- | --- | --- | --- |
|  |  | HR (95%CI) | *p* | HR (95%CI) | *p* |
| Clinical T stage | cT1 | Ref |  | Ref |  |
|  | cT2 | 0.88(0.26-3.02) | 0.84 |  |  |
|  | cT3 | 1.89(0.55-6.49) | 0.31 |  |  |
|  | cT4 | 2.82(0.78-10.25) | 0.12 |  |  |
| Clinical N stage | cN0 | Ref |  | Ref |  |
|  | cN1 | 2.22(0.85-5.8) | 0.10 | 1.88(0.69-5.09) | 0.22 |
|  | cN2 | 3.31(0.89-12.33) | 0.07 | 2.92(0.74-11.46) | 0.13 |
|  | cN3 | 5.01(1.74-14.43) | 0.001 | 1.67(0.51-5.48) | 0.40 |
| Age | <35 | Ref |  | Ref |  |
|  | 35-65 | 0.86(0.34-2.19) | 0.75 |  |  |
|  | >65 | 2.2(0.64-7.6) | 0.21 |  |  |
| BMI | BMI | 0.95(0.5-1.78) | 0.87 |  |  |
| Menopausal status | yes vs. no | 1.24(0.68-2.27) | 0.48 |  |  |
| Pathological T stage | pT0 | Ref |  | Ref |  |
|  | pTis | 0.5(0.06-3.99) | 0.51 | 0.47(0.06-3.87) | 0.48 |
|  | pT1 | 2.75(1.18-6.36) | 0.02 | 2.22(0.86-5.73) | 0.10 |
|  | pT2 | 7.2(3.1-16.7) | 0.001 | 3.37(1.19-9.54) | 0.02 |
|  | pT3/4 | 9.45(2-44.58) | 0.01 | 2.6(0.46-14.68) | 0.28 |
| Pathological N stage | pN0 | Ref |  | Ref |  |
|  | pN1 | 2.42(1.05-5.6) | 0.04 | 1.41(0.57-3.5) | 0.46 |
|  | pN2 | 6.41(2.85-14.44) | 0.001 | 3.1(1.2-8.03) | 0.02 |
|  | pN3 | 21.25(9.63-46.86) | 0.001 | 9.61(3.55-26.05) | 0.001 |
| Postoperative pathological type | No invasive tumor | Ref |  | Ref |  |
|  | DCIS | 0.5(0.06-3.99) | 0.51 | 0.76(0.09-6.58) | 0.81 |
|  | IDC | 4(1.82-8.78) | 0.001 | 3.35(1.31-8.54) | 0.01 |
|  | IDC+DCIS | 4.5(1.69-12.02) | 0.001 | 5.01(1.6-15.73) | 0.01 |
| pCR* | pCR* | 0.09(0.03-0.29) | 0.001 | 0.08(0.03-0.27) | 0.001 |
| ER | ER | 0.83(0.45-1.52) | 0.54 |  |  |
| PR | PR | 0.62(0.32-1.23) | 0.17 |  |  |
| HER2 | her2 3+ | Ref |  | Ref |  |
|  | her2 2+ | 1.63(0.73-3.66) | 0.24 | 1.03(0.44-2.39) | 0.95 |
|  | her2 1+ | 4.46(1.07-18.56) | 0.04 | 2.75(0.62-12.27) | 0.19 |
| Ki67 | ≥20% vs. <20% | 0.56(0.24-1.32) | 0.18 |  |  |
| Breast Surgery | Mastectomy vs. BCS | 0.12(0.02-0.88) | 0.04 | 0.18(0.02-1.33) | 0.09 |
| Axillary surgery | ALND | Ref |  | Ref |  |
|  | SLNB | 0(0-5.13E+282) | 0.97 |  |  |
|  | SLNB-ALND | 0.73(0.29-1.85) | 0.51 |  |  |
| Endocrine therapy | yes vs. no | 0.67(0.36-1.26) | 0.21 |  |  |
| Radiotherapy | yes vs. no | 0.8(0.4-1.58) | 0.52 |  |  |
| Adjuvant chemotherapy | yes vs. no | 1.65(0.88-3.11) | 0.12 |  |  |

| All variables were taken account into the multivariable Cox logistic analysis, including clinical T, N, age, BMI, menopausal status, preoperative ER, PR, HER2+, Ki67, surgery, axillary surgery, pT, pN, radiotherapy, endocrine therapy, adjuvant chemotherapy and trastuzumab. |
| --- |
| # pathological types were adjusted for clinical T, N, age, BMI, menopausal status, preoperative ER, PR, HER2+, Ki67, surgery, axillary surgery, pN, radiotherapy, endocrine therapy, adjuvant chemotherapy and trastuzumab |
| *pCR was adjusted for clinical T, N, age, BMI, menopausal status, preoperative ER, PR, HER2+, Ki67, surgery, axillary surgery, radiotherapy, endocrine therapy, adjuvant chemotherapy and trastuzumab |

Supplemental table 7: Cox proportional hazards regression analysis of DFS for the training cohort among those who received neoadjuvant wPC and trastuzumab

| Variables | Categories | Univariate COX analysis | | Multivariate COX analysis | |
| --- | --- | --- | --- | --- | --- |
|  |  | HR (95%CI) | *p* | HR (95%CI) | *p* |
| Clinical T stage | cT1 | Ref |  | Ref |  |
|  | cT2 | 1.01(0.3-3.46) | 0.982 |  |  |
|  | cT3 | 2(0.59-6.81) | 0.27 |  |  |
|  | cT4 | 2.62(0.72-9.51) | 0.144 |  |  |
| Clinical N stage | cN0 | Ref |  | Ref |  |
|  | cN1 | 2.08(0.92-4.72) | 0.081 |  |  |
|  | cN2 | 1.42(0.29-6.82) | 0.664 |  |  |
|  | cN3 | 2.62(0.92-7.48) | 0.072 |  |  |
| Age | <35 | Ref |  | Ref |  |
|  | 35-65 | 1.19(0.43-3.34) | 0.736 |  |  |
|  | >65 | 1.26(0.28-5.61) | 0.765 |  |  |
| BMI | Obesity vs. normal | 0.83(0.44-1.55) | 0.56 |  |  |
| Menopausal status | yes vs. no | 1.24(0.67-2.3) | 0.486 |  |  |
| Pathological T stage | pT0 | Ref |  | Ref |  |
|  | pTis | 0.94(0.27-3.26) | 0.916 | 0.77(0.22-2.78) | 0.692 |
|  | pT1 | 2.1(1.05-4.19) | 0.035 | 1.45(0.67-3.12) | 0.346 |
|  | pT2 | 3.9(1.77-8.61) | 0.001 | 2.06(0.79-5.39) | 0.14 |
|  | pT3/4 |  |  |  |  |
| Pathological N stage | pN0 | Ref |  | Ref |  |
|  | pN1 | 2.17(1.05-4.49) | 0.036 | 1.6(0.71-3.6) | 0.26 |
|  | pN2 | 4.68(2.08-10.53) | 0.001 | 3.69(1.49-9.15) | 0.005 |
|  | pN3 | 21.19(7.87-57.01) | 0.001 | 13.65(4.55-40.99) | 0.001 |
| Postoperative pathological type(#) | No invasive tumor | Ref |  | Ref |  |
|  | DCIS | 0.94(0.27-3.25) | 0.916 | 0.82(0.21-3.13) | 0.767 |
|  | IDC | 2.49(1.29-4.79) | 0.006 | 1.9(0.84-4.3) | 0.124 |
|  | IDC+DCIS | 2.73(0.98-7.58) | 0.054 | 1.94(0.61-6.18) | 0.264 |
| pCR* | yes vs. no | 0.27(0.13-0.54) | 0.001 | 0.26(0.12-0.54) | 0.001 |
| ER | Positive vs. negative | 1.82(1.02-3.23) | 0.042 | 1.1(0.57-2.12) | 0.774 |
| PR | Positive vs. negative | 1.34(0.75-2.4) | 0.326 |  |  |
| Her2 | 3+ | Ref |  | Ref |  |
|  | 2+ | 1.39(0.59-3.28) | 0.45 |  |  |
|  | 1+ | 0(0-1.51E+231) | 0.971 |  |  |
| Ki67 | ≥20% vs. <20% | 0.92(0.29-2.97) | 0.893 |  |  |
| Breast Surgery | Mastectomy vs. BCS | 0.6(0.22-1.68) | 0.334 |  |  |
| Axillary surgery | ALND | Ref |  | Ref |  |
|  | SLNB | 0.24(0.03-1.76) | 0.161 |  |  |
|  | SLNB-ALND | 0.99(0.44-2.21) | 0.977 |  |  |
| Endocrine therapy | yes vs. no | 1.35(0.76-2.41) | 0.312 |  |  |
| Radiotherapy | yes vs. no | 0.78(0.41-1.52) | 0.469 |  |  |
| Adjuvant chemotherapy | yes vs. no | 1.03(0.58-1.84) | 0.911 |  |  |

Supplemental table 8: Cox proportional hazards regression analysis of OS for the training cohort among those who received neoadjuvant wPC and trastuzumab

| Variables | Categories | Univariate COX analysis | | Multivariate COX analysis | |
| --- | --- | --- | --- | --- | --- |
|  |  | HR (95%CI) | *p* | HR (95%CI) | *p* |
| Clinical T stage | cT1 | Ref |  | Ref |  |
|  | cT2 | 1.01(0.3-3.46) | 0.982 |  |  |
|  | cT3 | 2(0.59-6.81) | 0.27 |  |  |
|  | cT4 | 2.62(0.72-9.51) | 0.144 |  |  |
| Clinical N stage | cN0 | Ref |  | Ref |  |
|  | cN1 | 2.08(0.92-4.72) | 0.081 |  |  |
|  | cN2 | 1.42(0.29-6.82) | 0.664 |  |  |
|  | cN3 | 2.62(0.92-7.48) | 0.072 |  |  |
| Age | <35 | Ref |  | Ref |  |
|  | 35-65 | 1.19(0.43-3.34) | 0.736 |  |  |
|  | >65 | 1.26(0.28-5.61) | 0.765 |  |  |
| BMI | BMI | 0.83(0.44-1.55) | 0.56 |  |  |
| Menopausal status | yes vs. no | 1.24(0.67-2.3) | 0.486 |  |  |
| Pathological T stage | pT0 | Ref |  | Ref |  |
|  | pTis | 0.94(0.27-3.26) | 0.916 | 0.77(0.21-2.76) | 0.686 |
|  | pT1 | 2.1(1.05-4.19) | 0.035 | 1.47(0.68-3.15) | 0.325 |
|  | pT2 | 3.9(1.77-8.61) | 0.001 | 2.13(0.83-5.43) | 0.115 |
|  | pT3/4 |  |  |  |  |
| Pathological N stage | pN0 | Ref |  | Ref |  |
|  | pN1 | 2.17(1.05-4.49) | 0.036 | 1.63(0.74-3.62) | 0.228 |
|  | pN2 | 4.68(2.08-10.53) | 0.001 | 3.83(1.6-9.18) | 0.003 |
|  | pN3 | 21.19(7.87-57.01) | 0.001 | 13.95(4.69-41.44) | 0.001 |
| Postoperative pathological type | No invasive tumor | Ref |  | Ref |  |
|  | DCIS | 0.94(0.27-3.25) | 0.916 | 0.85(0.22-3.32) | 0.82 |
|  | IDC | 2.49(1.29-4.79) | 0.006 | 2(0.86-4.64) | 0.107 |
|  | IDC+DCIS | 2.73(0.98-7.58) | 0.054 | 1.31(0.34-5.15) | 0.696 |
| pCR* | pCR* | 0.27(0.13-0.54) | 0.0001 | 0.25(0.11-0.54) | 0.0001 |
| ER | ER | 1.82(1.02-3.23) | 0.042 |  |  |
| PR | PR | 1.34(0.75-2.4) | 0.326 |  |  |
| HER2 | her2 3+ | Ref |  | Ref |  |
|  | her2 2+ | 1.39(0.59-3.28) | 0.45 |  |  |
|  | her2 1+ |  |  |  |  |
| Ki67 | ≥20% vs. <20% | 0.92(0.29-2.97) | 0.893 |  |  |
| Breast Surgery | Mastectomy vs. BCS | 0.6(0.22-1.68) | 0.334 |  |  |
| Axillary surgery | ALND | Ref |  | Ref |  |
|  | SLNB | 0.24(0.03-1.76) | 0.161 |  |  |
|  | SLNB-ALND | 0.99(0.44-2.21) | 0.977 |  |  |
| Endocrine therapy | yes vs. no | 1.35(0.76-2.41) | 0.312 |  |  |
| Radiotherapy | yes vs. no | 0.78(0.41-1.52) | 0.469 |  |  |
| Adjuvant chemotherapy | yes vs. no | 1.03(0.58-1.84) | 0.911 |  |  |

| All variables were taken account into the multivariable Cox logistic analysis, including clinical T, N, age, BMI, menopausal status, preoperative ER, PR, HER2+, Ki67, surgery, axillary surgery, pT, pN, radiotherapy, endocrine therapy, adjuvant chemotherapy and trastuzumab. |
| --- |
| # pathological types were adjusted for clinical T, N, age, BMI, menopausal status, preoperative ER, PR, HER2+, Ki67, surgery, axillary surgery, pN, radiotherapy, endocrine therapy, adjuvant chemotherapy and trastuzumab |
| *pCR was adjusted for clinical T, N, age, BMI, menopausal status, preoperative ER, PR, HER2+, Ki67, surgery, axillary surgery, radiotherapy, endocrine therapy, adjuvant chemotherapy and trastuzumab |

Supplemental table 9 Logistic analysis for factors that affect pCR in the training cohort among those who received neoadjuvant trastuzumab

|  |  |  |  |  | Univariate logistic analysis | | multivariate logistic analysis | |
| --- | --- | --- | --- | --- | --- | --- | --- | --- |
| Variables | All | Non-  pCR | pCR | pCR(%) | OR（95% CI） | P-value | OR（95% CI） | P-value |
| cT1 | 42 | 20 | 22 | 52.38% |  |  |  |  |
| cT2 | 277 | 162 | 115 | 41.52% | 0.65(0.34-1.24) | 0.65 |  |  |
| cT3 | 129 | 70 | 59 | 45.74% | 0.77(0.38-1.54) | 0.77 |  |  |
| cT4 | 59 | 36 | 23 | 38.98% | 0.58(0.26-1.29) | 0.58 |  |  |
| cN0 | 127 | 72 | 55 | 43.31% |  |  |  |  |
| cN1 | 301 | 169 | 132 | 43.85% | 1.02(0.67-1.55) | 0.92 |  |  |
| cN2 | 30 | 17 | 13 | 43.33% | 1.00(0.45-2.23) | 1.00 |  |  |
| cN3 | 59 | 35 | 24 | 40.68% | 0.90(0.48-1.68) | 0.74 |  |  |
| <35 | 51 | 31 | 20 | 39.22% |  |  |  |  |
| 35-65 | 437 | 243 | 194 | 44.39% | 1.24(0.68-2.24) | 0.48 |  |  |
| >65 | 29 | 19 | 10 | 34.48% | 0.82(0.32-2.11) | 0.67 |  |  |
| Obesity (BMI >25 vs <25) | 358 | 201 | 157 | 43.85% |  |  |  |  |
|  | 159 | 92 | 67 | 42.14% | 0.93(0.64-1.36) | 0.72 |  |  |
| Menopausal status (yes vs. no) | 211 | 123 | 88 | 41.71% | 1.12(0.78-1.59) | 0.54 |  |  |
|  | 306 | 170 | 136 | 44.44% |  |  |  |  |
| ER (negative vs. positive) | 304 | 139 | 165 | 54.28% | 0.32(0.22-0.47) | <0.001 | 0.51(0.30-0.86) | 0.01 |
|  | 213 | 154 | 59 | 27.70% |  |  |  |  |
| PR (negative vs. positive) | 351 | 169 | 182 | 51.85% | 0.32(0.21-0.47) | <0.001 | 0.50(0.28-0.89) | 0.02 |
|  | 166 | 124 | 42 | 25.30% |  |  |  |  |
| Her2 3+ | 454 | 246 | 208 | 45.81% |  |  |  |  |
| Her2 2+ | 57 | 42 | 15 | 26.32% | 0.24(0.03-2.04) | 0.19 | 0.37(0.04-3.32) | 0.38 |
| Her2 1+ | 6 | 5 | 1 | 16.67% | 0.42(0.23-0.78) | 0.006 | 0.47(0.25-0.91) | 0.02 |
| Ki67 (<20% vs. ≥20%) | 45 | 32 | 13 | 28.89% |  |  |  |  |
|  | 472 | 261 | 211 | 44.70% | 1.99(1.02-3.89) | 0.04 | 1.72(0.84-3.53) | 0.14 |
| enough cycle (no vs. yes) | 249 | 153 | 96 | 38.55% |  |  |  |  |
|  | 268 | 140 | 128 | 47.76% | 1.46(1.03-2.07) | 0.04 | 1.54(1.03-2.31) | 0.04 |
| wPC | 333 | 172 | 161 | 48.35% |  |  |  |  |
| 3wPC/TC | 69 | 43 | 26 | 37.68% | 0.65(0.38-1.10) | 0.11 | 0.64(0.36-1.12) | 0.12 |
| AC-T/P | 57 | 37 | 20 | 35.09% | 0.58(0.32-1.04) | 0.07 | 0.59(0.31-1.11) | 0.10 |
| Other | 58 | 41 | 17 | 29.31% | 0.44(0.24-0.81) | 0.008 | 0.49(0.25-0.95) | 0.04 |

Supplemental table 10 Factors that affect pCR in the validation cohort among those who received neoadjuvant trastuzumab

| Variables |  | No. | Non- pCR | pCR | X^2^ | p |
| --- | --- | --- | --- | --- | --- | --- |
| T | cT1 | 13 | 4 | 9 | 7.87 | 0.10 |
|  | cT2 | 95 | 49 | 46 |  |  |
|  | cT3 | 34 | 19 | 15 |  |  |
|  | cT4 | 10 | 8 | 2 |  |  |
| N | cN0 | 12 | 4 | 8 | 3.32 | 0.35 |
|  | cN1 | 117 | 61 | 56 |  |  |
|  | cN2 | 5 | 2 | 3 |  |  |
|  | cN3 | 20 | 13 | 7 |  |  |
| ER | negative | 94 | 40 | 54 | 8.35 | 0.003 |
|  | positive | 60 | 40 | 20 |  |  |
| PR | negative | 110 | 50 | 60 | 6.50 | 0.01 |
|  | positive | 44 | 30 | 14 |  |  |
| HER2 | 1+ | 3 | 2 | 1 | 6.57 | 0.04 |
|  | 2+ | 34 | 24 | 10 |  |  |
|  | 3+ | 117 | 54 | 63 |  |  |
| Ki-67 | <20% | 9 | 7 | 2 | 2.56 | 0.11 |
|  | ≥20% | 145 | 73 | 72 |  |  |
| Chemotherapy | wPC | 109 | 57 | 52 | 6.56 | 0.09 |
|  | 3wPC/TC | 17 | 12 | 5 |  |  |
|  | AC-T/P | 24 | 8 | 16 |  |  |
|  | Other | 4 | 3 | 1 |  |  |
| All predefined cycles | no | 52 | 27 | 25 | 0.00 | 1.00 |
|  | yes | 102 | 53 | 49 |  |  |

Supplemental table 11 Logistic analysis for factors that affect pCR in the training cohort among those who received neoadjuvant wPC and trastuzumab

|  |  |  |  |  |  | Univariate logistic analysis | | multivariate logistic analysis | |
| --- | --- | --- | --- | --- | --- | --- | --- | --- | --- |
|  | Variables | All | Non-  pCR | pCR | pCR(%) | OR（95% CI） | P-value | OR（95% CI） | P-value |
| T | cT1 | 31 | 14 | 17 | 54.84% |  |  |  |  |
|  | cT2 | 168 | 90 | 78 | 46.43% | 0.71(0.33-1.54) | 0.39 |  |  |
|  | cT3 | 86 | 41 | 45 | 52.33% | 0.9(0.4-2.06) | 0.81 |  |  |
|  | cT4 | 42 | 26 | 16 | 38.10% | 0.51(0.2-1.3) | 0.158 |  |  |
| N | cN0 | 85 | 46 | 39 | 45.88% |  |  |  |  |
|  | cN1 | 196 | 98 | 98 | 50.00% | 1.18(0.71-1.97) | 0.526 |  |  |
|  | cN2 | 16 | 8 | 8 | 50.00% | 1.18(0.41-3.44) | 0.762 |  |  |
|  | cN3 | 36 | 20 | 16 | 44.44% | 0.94(0.43-2.07) | 0.885 |  |  |
| Age | <35 | 31 | 14 | 17 | 54.84% |  |  |  |  |
|  | 35-65 | 281 | 145 | 136 | 48.40% | 0.77(0.37-1.63) | 0.497 |  |  |
|  | >65 | 21 | 13 | 8 | 38.10% | 0.51(0.16-1.57) | 0.238 |  |  |
| BMI | ≥25 | 223 | 114 | 109 | 48.88% |  |  |  |  |
|  | <25 | 110 | 58 | 52 | 47.27% | 0.94(0.59-1.48) | 0.783 |  |  |
| Menopausal status | Yes | 116 | 60 | 56 | 48.28% |  |  |  |  |
|  | No | 217 | 112 | 105 | 48.39% | 1(0.64-1.58) | 0.985 |  |  |
| ER | Negative | 197 | 78 | 119 | 60.41% |  |  |  |  |
|  | Positive | 136 | 94 | 42 | 30.88% | 0.29(0.18-0.47) | 0.001 | 0.39(0.21-0.74) | 0.004 |
| PR | Negative | 224 | 96 | 128 | 57.14% |  |  |  |  |
|  | Positive | 109 | 76 | 33 | 30.28% | 0.33(0.2-0.53) | 0.001 | 0.65(0.33-1.27) | 0.204 |
| HER2 | Her2 3+ | 296 | 147 | 149 | 50.34% |  |  |  |  |
|  | Her2 2+ | 35 | 23 | 12 | 34.29% | 0.52(0.25-1.07) | 0.076 |  |  |
|  | Her2 1+ | 2 | 2 | 0 | 0.00% |  |  |  |  |
| Ki67 | <20% | 20 | 14 | 6 | 30.00% |  |  |  |  |
|  | ≥20% | 313 | 158 | 155 | 49.52% | 2.29(0.86-6.11) | 0.098 |  |  |
| enough cycle | no | 153 | 85 | 68 | 44.44% |  |  |  |  |
|  | yes | 180 | 87 | 93 | 51.67% | 1.34(0.87-2.06) | 0.189 |  |  |

Supplemental table 12 Factors that affect pCR in the validation cohort among those who received neoadjuvant wPC and trastuzumab

| Variables |  | No. | Non- pCR | pCR | X^2^ | p |
| --- | --- | --- | --- | --- | --- | --- |
| T | cT1 | 6 | 1 | 5 |  |  |
|  | cT2 | 69 | 35 | 34 |  |  |
|  | cT3 | 26 | 15 | 11 |  |  |
|  | cT4 | 7 | 6 | 1 | 7.66 | 0.11 |
| N | cN0 | 6 | 0 | 6 |  |  |
|  | cN1 | 86 | 48 | 38 |  |  |
|  | cN2 | 3 | 1 | 2 |  |  |
|  | cN3 | 14 | 8 | 6 | 7.57 | 0.06 |
| ER | negative | 66 | 27 | 39 |  |  |
|  | positive | 43 | 30 | 13 | 8.69 | 0.003 |
| PR | negative | 78 | 34 | 44 |  |  |
|  | positive | 31 | 23 | 8 | 8.33 | 0.004 |
| HER2 | 1+ | 2 | 2 | 0 |  |  |
|  | 2+ | 24 | 20 | 4 |  |  |
|  | 3+ | 83 | 35 | 48 | 14.5 | 0.001 |
| Ki-67 | <20% | 5 | 4 | 1 |  |  |
|  | ≥20% | 104 | 53 | 51 | 1.61 | 0.2 |
| All predefined cycles | no | 41 | 20 | 21 |  |  |
|  | yes | 68 | 37 | 31 | 0.33 | 0.57 |

Supplemental table 13 The DEGs across three cohorts (74 genes)

|  |  | GSE37946 | | GSE50948 | | GSE66305 | |
| --- | --- | --- | --- | --- | --- | --- | --- |
|  |  | logFC | *p* | logFC | *p* | logFC | *p* |
| 58527 | ABRACL |  |  | 0.66 | 0.01 | 0.88 | 0.04 |
| AC128677.4 | AC128677.4 | 0.86 | 0.01 | 0.41 | 0.04 | 2.01 | 0.004 |
| AI694413 | AI694413 |  |  | 0.63 | 0.002 | 1.62 | 0.03 |
| AI915629 | AI915629 |  |  | 0.38 | 0.01 | 1.18 | 0.047 |
| 341 | APOC1 | 0.51 | 0.04 | 0.41 | 0.03 | 0.58 | 0.37 |
| BG548679 | BG548679 |  |  | 1.17 | 0.004 | 1.76 | 0.01 |
| 641 | BLM | 0.38 | 0.03 | 0.39 | 0.03 | 0.41 | 0.46 |
| 55299 | BRIX1 | 0.35 | 0.03 | 0.55 | 0.004 | 0.42 | 0.45 |
| 713 | C1QB | 0.69 | 0.03 | 0.50 | 0.03 | 0.85 | 0.15 |
| 84869 | CBR4 | 0.51 | 0.03 | 0.42 | 0.04 | 0.14 | 0.79 |
| 875 | CBS | 0.40 | 0.04 | 0.39 | 0.02 | 0.34 | 0.56 |
| 6362 | CCL18 | 0.46 | 0.04 | 0.45 | 0.07 | 1.86 | 0.02 |
| 6352 | CCL5 | 0.58 | 0.03 | 0.37 | 0.02 | 1.14 | 0.11 |
| 930 | CD19 | 0.58 | 0.03 | 0.40 | 0.03 | 1.20 | 0.05 |
| 914 | CD2 | 0.77 | 0.01 | 0.35 | 0.050 | 1.13 | 0.12 |
| 952 | CD38 | 0.46 | 0.04 | 0.33 | 0.049 | 1.28 | 0.06 |
| 80347 | COASY | 0.33 | 0.04 | 0.43 | 0.02 | -0.23 | 0.68 |
| 11151 | CORO1A | 0.77 | 0.02 | 0.49 | 0.01 | 0.71 | 0.24 |
| 79174 | CRELD2 | 0.35 | 0.03 | 0.34 | 0.04 | 0.27 | 0.60 |
| 1075 | CTSC | 0.52 | 0.047 | 0.32 | 0.07 | 0.99 | 0.04 |
| 3627 | CXCL10 | 0.67 | 0.02 | 0.67 | 0.02 | 1.72 | 0.01 |
| 6373 | CXCL11 | 0.53 | 0.03 | 0.44 | 0.03 | 1.80 | 0.02 |
| 10563 | CXCL13 | 1.29 | 0.01 | 0.76 | 0.01 | 2.60 | 0.003 |
| 4283 | CXCL9 | 0.70 | 0.07 | 0.89 | 0.01 | 1.75 | 0.02 |
| 9595 | CYTIP | 0.62 | 0.051 | 0.56 | 0.04 | 1.48 | 0.04 |
| 1933 | EEF1B2 | 0.61 | 0.00 | 0.45 | 0.02 | 0.35 | 0.47 |
| 3646 | EIF3E | 0.43 | 0.03 | 0.38 | 0.047 | 0.56 | 0.21 |
| 2173 | FABP7 | 0.72 | 0.04 | 0.65 | 0.03 | 0.28 | 0.74 |
| 81558 | FAM117A | 0.63 | 0.01 | 0.42 | 0.04 | 0.08 | 0.89 |
| 83416 | FCRL5 |  |  | 0.47 | 0.02 | 1.30 | 0.049 |
| 2289 | FKBP5 | 0.40 | 0.04 | 0.44 | 0.04 | 0.69 | 0.24 |
| 2633 | GBP1 | 0.68 | 0.04 | 0.43 | 0.04 | 1.15 | 0.02 |
| 2634 | GBP2 | 0.58 | 0.02 | 0.15 | 0.50 | 1.33 | 0.03 |
| 8836 | GGH | 0.18 | 0.46 | 0.70 | 0.001 | 1.32 | 0.02 |
| 51053 | GMNN | 0.44 | 0.06 | 0.73 | 0.01 | 0.91 | 0.03 |
| 1880 | GPR183 | 0.60 | 0.02 | 0.30 | 0.23 | 1.48 | 0.03 |
| 3003 | GZMK | 0.61 | 0.04 | 0.43 | 0.05 | 1.36 | 0.12 |
| 3120 | HLA-DQB2 | 0.45 | 0.03 | 0.34 | 0.05 | 0.60 | 0.46 |
| 6782 | HSPA13 | 0.08 | 0.69 | 0.54 | 0.01 | 0.90 | 0.04 |
| 3492 | IGH | 0.53 | 0.02 | 0.44 | 0.01 | 1.03 | 0.12 |
| 3507 | IGHM | 0.90 | 0.01 | 0.50 | 0.01 | 1.93 | 0.01 |
| 50802 | IGK | 0.61 | 0.04 | 0.49 | 0.02 | 1.79 | 0.02 |
| 3514 | IGKC | 0.73 | 0.02 | 0.61 | 0.01 | 1.70 | 0.01 |
| IGKV1-17 | IGKV1-17 | 1.18 | 0.01 | 0.85 | 0.01 | 3.01 | 0.0001 |
| IGKV1-37 | IGKV1-37 | 1.05 | 0.01 | 0.56 | 0.01 | 2.50 | 0.003 |
| IGKV1OR2-108 | IGKV1OR2-108 | 1.05 | 0.01 | 0.49 | 0.02 | 2.31 | 0.002 |
| 3537 | IGLC1 | 0.65 | 0.04 | 0.52 | 0.01 | 1.29 | 0.04 |
| 28831 | IGLJ3 | 0.70 | 0.01 | 0.60 | 0.001 | 1.64 | 0.02 |
| 91353 | IGLL3P | 0.50 | 0.09 | 0.63 | 0.004 | 1.24 | 0.04 |
| 1E+08 | IGLL5 | 0.59 | 0.04 | 0.54 | 0.005 | 2.24 | 0.01 |
| 28823 | IGLV1-44 | 0.52 | 0.053 | 0.45 | 0.01 | 1.64 | 0.02 |
| 3560 | IL2RB | 0.46 | 0.047 | 0.64 | 0.01 | 1.13 | 0.07 |
| 9235 | IL32 | 0.55 | 0.04 | 0.41 | 0.02 | 0.23 | 0.72 |
| 3689 | ITGB2 | 0.57 | 0.04 | 0.32 | 0.05 | 0.59 | 0.33 |
| 3936 | LCP1 | 0.60 | 0.03 | 0.51 | 0.04 | 0.58 | 0.29 |
| 4001 | LMNB1 | 0.36 | 0.048 | 0.48 | 0.02 | 0.45 | 0.34 |
| 1.02E+08 | LOC101929272 | 0.28 | 0.13 | 0.37 | 0.05 | 1.31 | 0.050 |
| 9404 | LPXN | 0.55 | 0.04 | 0.36 | 0.05 | 0.46 | 0.37 |
| 4069 | LYZ | 0.71 | 0.03 | 0.63 | 0.06 | 1.99 | 0.005 |
| M24668 | M24668 | 0.64 | 0.04 | 0.58 | 0.003 | 2.08 | 0.01 |
| 9833 | MELK | 0.18 | 0.34 | 0.61 | 0.000 | 1.05 | 0.02 |
| 55706 | NDC1 | 0.45 | 0.03 | 0.34 | 0.04 | 0.54 | 0.23 |
| 5450 | POU2AF1 | 1.00 | 0.02 | 0.41 | 0.05 | 1.13 | 0.10 |
| 54440 | SASH3 | 0.55 | 0.04 | 0.52 | 0.02 | 0.84 | 0.19 |
| 10507 | SEMA4D | 0.49 | 0.02 | 0.50 | 0.01 | 0.67 | 0.29 |
| 5265 | SERPINA1 | 0.53 | 0.01 | 0.48 | 0.004 | 0.11 | 0.86 |
| 55423 | SIRPG | 0.50 | 0.03 | 0.36 | 0.05 | 0.47 | 0.52 |
| 57823 | SLAMF7 | 0.43 | 0.03 | 0.69 | 0.01 | 1.10 | 0.11 |
| 54733 | SLC35F2 | 0.50 | 0.02 | 0.41 | 0.04 | 0.73 | 0.18 |
| 6646 | SOAT1 | 0.19 | 0.27 | 0.41 | 0.02 | 0.93 | 0.047 |
| 6664 | SOX11 | 0.20 | 0.59 | 0.93 | 0.002 | 2.53 | 0.01 |
| 11262 | SP140 | 0.50 | 0.05 | 0.44 | 0.02 | 1.30 | 0.04 |
| 51765 | STK26 | 0.56 | 0.03 | 0.56 | 0.01 | 0.17 | 0.71 |
| 54490 | UGT2B28 | 0.70 | 0.02 | 0.84 | 0.003 | 2.29 | 0.046 |

Supplemental table 14 Signatures for each set

| Set | Gene |
| --- | --- |
| B cell | CD19, CD38, HLADQB2, FCRL5, IGHM, IGKC, IGLC1, IGLL5, IGH, IGK, IGKV117, IGKV137, IGKV1OR2108, IGLJ3, IGLL3P, IGLV144, GPR183, LPXN, POU2AF1, SP140 |
| cell cycle | CYTIP, BLM, GMNN, MELK, SOX11, STK26 |
| Cell chemotaxis | CCL18, CXCL10, CXCL11, CXCL13, CXCL9 |
| Immune Set 1 | C1QB, CTSC, GGH, IL2RB, IL32, LCP1, LYZ |
| Metabolic | APOC1, CBR4, CBS, COASY, FABP7, SOAT1, UGT2B28 |
| NK cell | CD2, ITGB2, SLAMF7 |
| Immune Set 2 | ABRACL, BRIX1, CRELD2, EEF1B2, EIF3E, FAM117A, FKBP5, HSPA13, NDC1, SERPINA1, SLC35F2 |
| T cell | CCL5, CORO1A, GBP1, GBP2, GZMK, SASH3, SEMA4D, SIRPG |

Supplemental table 15 The signatures between pCR and non-pCR in different datasets

|  | GSE37946 | | | GSE 50948 | | | GSE 66305 | | | GSE 130788 | | |
| --- | --- | --- | --- | --- | --- | --- | --- | --- | --- | --- | --- | --- |
| Signature | pCR | Non-pCR | *p* | pCR | Non-pCR | *p* | pCR | Non-pCR | *p* | pCR | Non-pCR | *p* |
| B cell | 83.96±44.13 | 57.69±32.62 | 0.025 | 106.73±32.11 | 80.06±43.19 | 0.007 | 99.45±38.18 | 49.91±42.96 | 0.021 | 45.58±20.09 | 32.02±13.87 | 0.04 |
| cell cycle | 28.93±10.12 | 22.88±9.30 | 0.036 | 33.92±8.45 | 25.07±9.53 | 0.0002 | 34.59±6.88 | 20.58±8.95 | 0.002 | 37.11±11.73 | 31.78±7.27 | 0.15 |
| Cell chemotaxis | 22.26±9.49 | 15.97±8.15 | 0.018 | 22.08±6.31 | 16.66±8.46 | 0.006 | 38.62±8.41 | 23.26±11.66 | 0.008 | 29.56±11.58 | 20.32±7.08 | 0.01 |
| Immune Set 1 | 34.36±12.53 | 26.61±10.20 | 0.024 | 38.94±9.99 | 30.78±13.00 | 0.007 | 46.01±12.20 | 31.68±12.31 | 0.023 | 37.60±15.74 | 25.65±7.30 | 0.01 |
| Metabolic | 31.60±10.19 | 24.05±9.38 | 0.01 | 36.23±9.66 | 27.79±11.20 | 0.002 | 33.16±12.07 | 25.76±11.51 | 0.195 | 34.38±10.86 | 24.97±9.91 | 0.02 |
| NK cell | 14.68±5.71 | 10.85±4.89 | 0.017 | 17.42±5.08 | 13.75±6.31 | 0.014 | 12.91±4.83 | 8.74±6.72 | 0.179 | 17.50±8.12 | 12.04±4.80 | 0.03 |
| Immune Set 2 | 58.09±15.12 | 45.40±14.53 | 0.005 | 60.52±16.48 | 47.56±16.79 | 0.003 | 57.26±14.28 | 41.13±15.27 | 0.043 | 53.60±15.31 | 40.08±14.60 | 0.02 |
| T cell | 40.76±17.92 | 29.92±13.04 | 0.022 | 39.82±12.61 | 31.01±15.28 | 0.015 | 46.54±11.76 | 33.45±17.33 | 0.06 | 47.08±21.25 | 32.99±14.18 | 0.04 |
| Model | 16.63±7.02 | 11.26±5.54 | 0.01 | 18.89±5.03 | 13.74±7.09 | 0.002 | 25.55±7.66 | 13.48±7.61 | 0.003 | 16.17±6.39 | 11.46±4.27 | 0.02 |

Supplemental table 16 The pCR rates across different cutoff points

|  |  | Non-pCR | pCR | % | *p* | Univariate logistic analysis | Multivariate logistic analysis |
| --- | --- | --- | --- | --- | --- | --- | --- |
| GSE37946 | <10 | 10 | 4 | 28.6% |  |  |  |
|  | 10-19.99 | 10 | 15 | 60% |  |  |  |
|  | ≥20 | 2 | 8 | 80% | 0.035 | 1.15(1.03 1.28) | 1.18(1.04 1.33) |
| GSE 50948 | <10 | 11 | 2 | 15.4% |  |  |  |
|  | 10-19.99 | 14 | 15 | 51.7% |  |  |  |
|  | ≥20 | 7 | 14 | 66.7% | 0.014 | 1.15(1.05 1.26) | 1.13(1.03 1.25) |
| GSE 66305 | <10 | 6 | 0 | 0% |  |  |  |
|  | 10-19.99 | 8 | 2 | 20% |  |  |  |
|  | ≥20 | 3 | 4 | 57.1% | 0.05 | 1.20(1.03 1.41) |  |
| GSE 130788 | <10 | 3 | 1 | 25% |  |  |  |
|  | 10-19.99 | 10 | 5 | 33.33% |  |  |  |
|  | ≥20 | 3 | 9 | 75% | 0.05 | 1.19(1.01 1.40) | 1.19(1.00 1.42) |

Supplemental table 17 The signatures between HR+ and HR- in GSE50948

| Signature | ER+ | ER- | *p* | PR+ | PR- | *p* | ER+PR+ | ER-PR- | *p* |
| --- | --- | --- | --- | --- | --- | --- | --- | --- | --- |
| B cell | 78.83±46.28 | 97.67±37.45 | 0.11 | 68.05±42.5 | 99.1±37.61 | 0.015 | 64.11±41.81 | 100.47±35.7 | 0.018 |
| cell cycle | 25.38±12.06 | 30.69±9.03 | 0.07 | 21.21±10.18 | 31.36±9 | 0.001 | 18.05±9.69 | 31.28±8.84 | 0.001 |
| Cell chemotaxis | 16.38±9.09 | 20.24±7.37 | 0.1 | 12.74±7.62 | 20.87±7.2 | 0.001 | 12.99±8.7 | 21.16±6.94 | 0.008 |
| Immune Set 1 | 31.16±12.66 | 35.93±12.01 | 0.19 | 24.57±10.46 | 37.2±11.42 | 0.001 | 24.18±11.14 | 37.19±11.61 | 0.008 |
| Metabolic | 28.13±11.71 | 33.14±10.92 | 0.13 | 24.6±10.54 | 33.67±10.76 | 0.011 | 22.5±11.4 | 33.79±10.98 | 0.015 |
| NK cell | 14.34±6.99 | 15.94±5.66 | 0.37 | 11.3±6.36 | 16.56±5.49 | 0.005 | 11.26±6.47 | 16.47±5.34 | 0.024 |
| Immune Set 2 | 51.09±21.98 | 54.83±16.38 | 0.48 | 41.9±18.19 | 56.77±16.58 | 0.008 | 37.78±17.82 | 55.66±16.09 | 0.01 |
| T cell | 33.07±16.5 | 36.05±14.08 | 0.49 | 25.46±13.52 | 37.67±13.98 | 0.008 | 25.72±14.48 | 37.33±13.71 | 0.045 |
| Model | 13.73±7.29 | 17.07±6.29 | 0.09 | 12.32±6.67 | 17.21±6.34 | 0.02 | 12.08±6.53 | 17.59±6.02 | 0.03 |

Supplemental table 18 The signatures between HR+ and HR- in GSE130788

| Signature | ER+ | ER- | *p* | PR+ | PR- | *p* | ER+PR+ | ER-PR- | *p* |
| --- | --- | --- | --- | --- | --- | --- | --- | --- | --- |
| B cell | 34±18.34 | 43.46±17.37 | 0.15 | 37.71±17.46 | 39.06±19.06 | 0.85 | 34.73±15.17 | 41.74±16.65 | 0.3 |
| cell cycle | 30.98±7.23 | 37.96±11.27 | 0.05 | 31.68±8.63 | 35.83±10.44 | 0.27 | 30.36±7.85 | 37.47±11.52 | 0.11 |
| Cell chemotaxis | 19.8±8.97 | 30.12±9.49 | 0.004 | 21.91±10.92 | 26.38±10.15 | 0.26 | 19.31±7.07 | 28.85±8.42 | 0.01 |
| Immune Set 1 | 26.38±11.34 | 36.81±13.63 | 0.03 | 29.61±14.82 | 32.43±12.82 | 0.58 | 25.88±8.55 | 34.66±11.19 | 0.049 |
| Metabolic | 24.52±9.93 | 34.86±10.36 | 0.01 | 24.88±13.01 | 32.07±9.6 | 0.09 | 22±9.28 | 33.51±9.29 | 0.01 |
| NK cell | 12.45±6.79 | 17.07±6.77 | 0.07 | 13.86±6.62 | 15.13±7.43 | 0.64 | 12.37±4.63 | 16.23±6.16 | 0.11 |
| Immune Set 2 | 42.03±15.44 | 51.52±16.09 | 0.11 | 42.79±18.34 | 48.73±15.01 | 0.34 | 40.24±17.14 | 50.31±15.98 | 0.15 |
| T cell | 34.61±18 | 45.36±19.13 | 0.12 | 38.29±17.81 | 40.64±20.08 | 0.75 | 34.34±12.74 | 43.04±17.54 | 0.2 |
| Model | 10.94±4.35 | 16.74±5.82 | 0.004 | 12.23±6.71 | 14.58±5.27 | 0.29 | 10.53±3.88 | 15.85±4.87 | 0.01 |

Supplemental table 19 The signatures between HR+ and HR- in GSE37946

| Signature | ER+ | ER- | *p* | PR+ | PR- | *p* | ER+PR+ | ER-PR- | *p* |
| --- | --- | --- | --- | --- | --- | --- | --- | --- | --- |
| B cell | 69.03±45.26 | 73.99±39.27 | 0.69 | 54.39±37.63 | 78.59±40.98 | 0.07 | 55.91±38.89 | 75.25±39.29 | 0.16 |
| cell cycle | 23.95±10.18 | 27.53±10.02 | 0.24 | 21.69±9.6 | 27.85±9.93 | 0.06 | 21.71±10.03 | 27.73±10.13 | 0.09 |
| Cell chemotaxis | 19.34±11.29 | 19.49±8.26 | 0.96 | 20.41±8.06 | 16.75±12.3 | 0.23 | 17.33±12.66 | 19.81±8.2 | 0.45 |
| Immune Set 1 | 29.95±13.83 | 31.43±11.13 | 0.68 | 32.51±11.28 | 26.39±13.49 | 0.12 | 26.69±14.05 | 31.72±11.2 | 0.23 |
| Metabolic | 25.73±9.92 | 29.65±10.63 | 0.21 | 29.83±10.64 | 23.73±8.74 | 0.07 | 23.49±9.08 | 29.76±10.79 | 0.08 |
| NK cell | 11.94±6.33 | 13.56±5.22 | 0.34 | 13.9±5.34 | 10.35±5.84 | 0.05 | 10.33±6.1 | 13.65±5.28 | 0.09 |
| Immune Set 2 | 49.25±16.34 | 54.22±15.82 | 0.3 | 54.77±15.68 | 45.82±15.69 | 0.08 | 46.1±16.36 | 54.61±15.93 | 0.13 |
| T cell | 34.87±20.07 | 36.48±14.68 | 0.75 | 38.33±15.36 | 29.14±18.9 | 0.09 | 29.74±19.61 | 36.97±14.68 | 0.2 |
| Model | 14.02±8.23 | 14.34±6.12 | 0.88 | 13±11.39 | 36±15.25 | 0.11 | 11.68±7.48 | 14.56±6.1 | 0.2 |

Supplemental table 20 The signatures between HR+ and HR- in GSE58984

| Signature | ER+ | ER- | *p* | PR+ | PR- | *p* | ER+PR+ | ER-PR- | *p* |
| --- | --- | --- | --- | --- | --- | --- | --- | --- | --- |
| B cell | 89.88±38.11 | 89.83±35.6 | 1 | 90.96±36.82 | 88.76±37.6 | 0.77 | 90.41±37.02 | 89.02±35.83 | 0.87 |
| cell cycle | 31.51±6.93 | 32.4±8.56 | 0.58 | 31±6.75 | 32.66±8.22 | 0.29 | 30.8±6.68 | 32.17±8.58 | 0.43 |
| Cell chemotaxis | 28.36±9.93 | 27.98±10.02 | 0.86 | 28.66±9.98 | 27.78±9.93 | 0.67 | 28.53±10.05 | 27.78±10.11 | 0.74 |
| Immune Set 1 | 37.47±12.24 | 34.98±10.45 | 0.32 | 37.4±11.77 | 35.75±11.55 | 0.49 | 37.31±11.88 | 34.78±10.54 | 0.33 |
| Metabolic | 31.38±8.24 | 32.53±7.85 | 0.51 | 30.26±8.48 | 33.33±7.43 | 0.07 | 30.32±8.56 | 32.68±7.92 | 0.22 |
| NK cell | 16.34±6.51 | 15.47±6.26 | 0.53 | 16.27±6.26 | 15.77±6.6 | 0.7 | 16.24±6.32 | 15.39±6.34 | 0.56 |
| Immune Set 2 | 64.03±10.8 | 60.26±12.91 | 0.13 | 63.15±10.52 | 62.18±12.84 | 0.69 | 63.06±10.62 | 60.04±13.05 | 0.26 |
| T cell | 40.18±14.2 | 36.72±13.8 | 0.25 | 39.78±13.1 | 38.08±15.09 | 0.56 | 39.65±13.22 | 36.45±13.92 | 0.3 |
| Model | 18.42±6.84 | 18.85±6.23 | 0.76 | 18.4±6.61 | 18.74±6.65 | 0.81 | 18.32±6.65 | 18.74±6.3 | 0.78 |

Supplmental materials

**Clinical models for those who received neoadjuvant trastuzumab among HER2+ breast cancer patients**

y1=-0.695×ER-0.683×PR+0.688×HER2-0.210×chemotherapy+0.388×all-cycle-1.61 (Figure 2, supplemental materials).

ER: ER negative was defined as “0”, and ER positive was defined as “1”.

PR: PR negative was defined as “0”, and PR positive was defined as “1”.

HER2: IHC 1 was defined as “1”, IHC 2 was defined as “2”, IHC 3 was defined as “3”.

Chemotherapy: weekly paclitaxel and carboplatin (wPC) was defined as “1”, Paclitaxel and carboplatin administrated thrice weekly (3wPC) was defined as “2”, anthracycline followed taxanes (AC-T/P) was defined as “3”, anthracycline+taxanes regimen (TAC) was defined as “4”, and other regimens were defined as “5”.

all-cycle: Completion of all predefined cyclyes was defined as “1” and otherwises were defined as “0”.

Those who received neoadjuvant trastuzumab or wPCH could be regarded as a specific case and the trastuzumab was defined as 1 and/or the chemotherapy was defined as 1.

**Clinical models for HER2+ breast cancer patients in neoadjuvant setting**

y1=-0.698×ER-0.626×PR+0.742×HER2+0.926×Trastuzumab-0.236×chemotherapy+0.387×all-cycle-2.66

Trastuzumab: without trastuzumab was defined as “0”, and trastuzumab use was defined as “1”.

ER: ER negative was defined as “0”, and ER positive was defined as “1”.

PR: PR negative was defined as “0”, and PR positive was defined as “1”.

HER2: IHC 1 was defined as “1”, IHC 2 was defined as “2”, IHC 3 was defined as “3”.

Chemotherapy: weekly paclitaxel and carboplatin (wPC) was defined as “1”, Paclitaxel and carboplatin administrated thrice weekly (3wPC) was defined as “2”, anthracycline followed taxanes (AC-T/P) was defined as “3”, anthracycline+taxanes regimen (TAC) was defined as “4”, and other regimens were defined as “5”.

all-cycle: Completion of all predefined cyclyes was defined as “1” and otherwises were defined as “0”.

Those who received neoadjuvant trastuzumab or wPCH could be regarded as a specific case and the trastuzumab was defined as 1 and/or the chemotherapy was defined as 1.


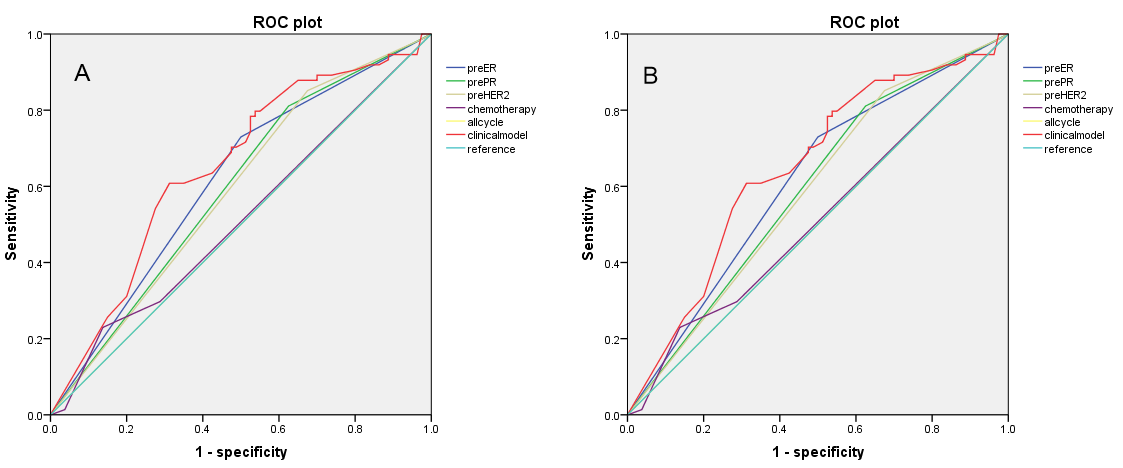


Supplemental Figure 1 The clinical model in the training cohort (A) and validation cohort (B)


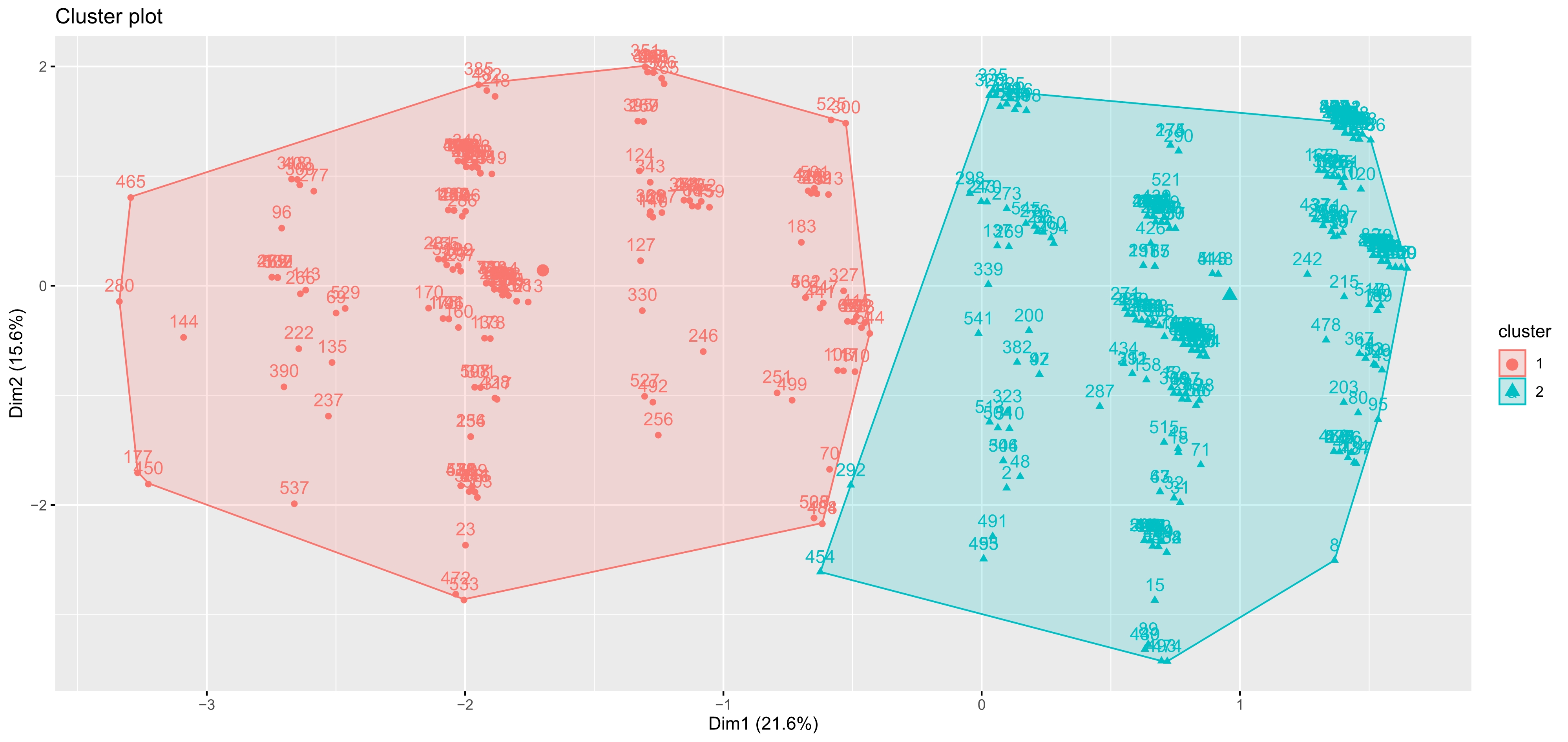


Supplemental Figure 2 cluster analysis of the patients in the training cohort
